# The electrophilic metabolite of kynurenine, kynurenine-CKA, requires C151 in Keap1 to derepress Nrf2

**DOI:** 10.1016/j.redox.2026.104009

**Published:** 2026-01-06

**Authors:** Jialin Feng, Mara Carreño, Hannah Jung, Sharadha Dayalan Naidu, Nicole Arroyo-Diaz, Abel D. Ang, Bhargavi Kulkarni, Dorothy Kisielewski, Takafumi Suzuki, Masayuki Yamamoto, John D. Hayes, Tadashi Honda, Landon Wilson, Beatriz Leon-Ruiz, Aimee L. Eggler, Dario A. Vitturi, Albena T. Dinkova-Kostova

**Affiliations:** a Jacqui Wood Cancer Centre, Division of Cancer Research, School of Medicine, University of Dundee, Dundee, UK; b Department of Pathology, School of Medicine, The University of Alabama at Birmingham, Birmingham, AL, USA; c Department of Chemistry and Biochemistry, Villanova University, Villanova, PA, USA; d Department of Biochemistry and Molecular Biology, Tohoku Medical Megabank Organization, Tohoku University, Sendai, Japan; e Department of Chemistry and Institute of Chemical Biology & Drug Discovery, Stony Brook University, Stony Brook, NY, USA; f Targeted Metabolomics and Proteomics Laboratory, Department of Pharmacology and Toxicology, University of Alabama at Birmingham, AL, USA; g Innate Cells and Th2 Immunity Section, Laboratory of Allergic Diseases, National Institute of Allergy and Infectious Diseases, National Institutes of Health, Bethesda, MD, USA; h Department of Physiology, Pharmacology and Therapeutics and Department of Medicine, Johns Hopkins University, School of Medicine, Baltimore, MD, USA

## Abstract

The Kelch-like ECH-associated protein 1/Nuclear factor-erythroid 2 p45-related factor 2 (Keap1/Nrf2) system responds to a wide array of structurally diverse small molecules, of both exogenous and endogenous origin, by inducing a robust cytoprotective program that allows adaptation during oxidative, metabolic and inflammatory stress. Here, we report that exposure to the tryptophan metabolite kynurenine and its electrophilic derivative kynurenine-carboxyketoalkene (Kyn-CKA) leads to an increase in the abundance of transcription factor Nrf2 and induction of Nrf2-target genes, including NAD(P)H:quinone oxidoreductase 1 (NQO1), in murine and human cells. Additionally, both kynurenine and Kyn-CKA activate the aryl hydrocarbon receptor (AhR). Using cellular thermal shift assays, we found that Kyn-CKA increases the thermal stability of Keap1-mCherry fusion protein, but not free mCherry, indicating target engagement of Keap1, the principal repressor of Nrf2. Critically, the ability of Kyn-CKA to increase the abundance of Nrf2 and expression of NQO1 in mouse embryonic fibroblasts (MEFs) expressing wild-type Keap1 was greatly diminished in C151S-Keap1 mutant MEFs. Furthermore, Kyn-CKA reacts with Keap1 C151 much faster *in vitro* than with the small molecule thiol *N*-acetyl cysteine, suggesting that Kyn-CKA is targeted to C151 by the surrounding active site. Experiments in wild-type, AhR-knockout, and Nrf2-knockout primary murine bone marrow-derived macrophages showed that Nrf2 is required for the acute anti-inflammatory activity of Kyn-CKA, whereas AhR is dispensable. Together, these findings demonstrate that Kyn-CKA requires C151 in Keap1 to derepress Nrf2 and reveal that Nrf2, but not AhR, is a main contributor to the anti-inflammatory activity of Kyn-CKA in macrophages.

## 1 Introduction

In mammalian cells, the Kelch-like ECH-associated protein 1/Nuclear factor-erythroid 2 p45-related factor 2 (Keap1/Nrf2) system is the main orchestrator of the cellular defence against environmental stress, most notably oxidative and inflammatory stress [1]. In this partnership, transcription factor Nrf2 is constitutively repressed by the activity of several ubiquitin ligase systems that promote its ubiquitination and subsequent proteasomal degradation, and Keap1, a substrate adaptor protein for the cullin 3 (Cul3)-really interesting new gene (RING) ubiquitin ligase complex, represents the principal negative regulator of Nrf2 [2]. During cellular stress, such as oxidative or electrophilic stress, Keap1 loses its repressor activity, and consequently *de novo* synthesized Nrf2 accumulates in the nucleus. This enables Nrf2 to heterodimerize with a small Maf protein and bind to the regulatory regions of genes that contain antioxidant/electrophile-responsive element (ARE/EpRE; i.e., 5′-TGAC/GNNNGC-3′) sequences, inducing a cytoprotective transcriptional program, which restores redox homeostasis. Prominent among the transcriptional targets of Nrf2 are genes encoding antioxidant enzymes, such as NAD(P)H:quinone oxidoreductase 1 (NQO1) and both the catalytic (GCLC) and the regulatory (GCLM) subunits of glutamate-cysteine ligase, which together catalyze the rate-limiting step in the biosynthesis of glutathione, the principal intracellular antioxidant. Additionally, Nrf2 activation has anti-inflammatory effects in cells, mice and humans, by suppressing the expression of pro-inflammatory genes, and thus plays a critical role in the resolution of inflammation [[3], [4], [5]].

Nrf2 can be activated pharmacologically by small molecules, the majority of which are electrophiles and oxidants that modify specific cysteine-based sensors in Keap1 [6,7]. Being in proximity to basic amino acids [8], C151 located in the Broad complex, Tramtrack and Bric-à-Brac (BTB) homodimerization domain of Keap1 (residues 51–180 [2]) is particularly reactive at physiological pH (p*K*_a_ = 6.9) [9], and recent work has revealed that, through mostly hydrophobic interactions, the reaction of C151 with structurally diverse Nrf2 activators is catalytic, with a key hydrogen bond orienting their electrophilic carbons within ∼3–5 Å of C151 for a catalytic proximity effect [9]. This is in full agreement with findings from mutagenesis studies showing that this cysteine is the main intracellular target for the most potent chemical class of inducers, the cyanoenones, irrespective of their molecular size and shape [10]. Of the 11 Keap1 sensor cysteines, C151 is the target of the largest number of known Nrf2 activators. Almost all α,β-unsaturated carbonyl Nrf2 activators preferentially react with Keap1 C151 and require C151 to activate Nrf2, and the cyanoenones are a particularly potent subclass. Notably, C151 in Keap1 is the target of the two Nrf2 activators that are in clinical use: dimethyl fumarate, for relapsing remitting multiple sclerosis, and omaveloxolone, for Friedreich's ataxia, as well as the isothiocyanate sulforaphane, a classical Nrf2 activator that has been employed in ∼90 clinical trials [[11], [12], [13]].

Numerous electrophilic Nrf2 activators belonging to several distinct chemical classes have been shown to suppress pro-inflammatory responses, with a linear correlation between their NQO1-inducer and inducible nitric acid synthase (iNOS)-inhibitory potencies spanning more than six orders of magnitude of concentrations [14,15]. Such striking correlation strongly suggests that, although the Keap1/Nrf2 system is not their only cellular target, it represents a significant contributor to the anti-inflammatory effects of these compounds. Most Nrf2 activators are xenobiotics, but it is becoming increasingly clear that several endogenously produced metabolites, particularly those that have electrophilic properties, can also react with cysteine-based sensors of Keap1 to activate Nrf2 [16].

Kynurenine is an endogenous metabolite derived from the essential amino acid tryptophan. It has recently been identified as a major metabolite within the tumour microenvironment, especially in aggressive cancers such as glioblastoma multiforme (GBM), where kynurenine is present at micromolar concentrations and promotes the establishment of an anti-inflammatory phenotype of tumour-associated macrophages, facilitating tumor growth [17]. Study of tumour-associated macrophages showed that the anti-inflammatory effect of kynurenine is mediated, in part, by activation of the aryl hydrocarbon receptor (AhR), a ligand-activated transcription factor that triggers expression of genes involved in numerous biological processes, including drug metabolism, haematopoiesis, angiogenesis and immunity [18]. Crosstalk between AhR and Nrf2 has been described, where the two transcription factors influence each other's gene expression [19,20]. Notably, kynurenine and its metabolites, such as the electrophilic α,β-unsaturated carbonyl kynurenine-carboxyketoalkene (Kyn-CKA), have been demonstrated to activate Nrf2 in other pathologies, including sickle cell disease, attenuating inflammation [21]. Moreover, identification of the gene encoding the kynurenine-metabolising enzyme kynureninase as a gene transcriptionally upregulated by Nrf2 [22], provides a plausible negative feedback regulatory mechanism.

Because kynurenine is not electrophilic, whereas its metabolite Kyn-CKA is an α,β-unsaturated carbonyl, we considered the possibility that Kyn-CKA is the actual Nrf2 activator. Using biochemical assays, we found that, the reaction of Kyn-CKA with C151 in the BTB domain of the protein is many times faster than its reaction with *N*-acetyl cysteine (NAC), indicating that in cells, Kyn-CKA will target C151 much faster than other cysteines. In cell lysates, Kyn-CKA increases the thermostability of Keap1, indicating target engagement. Consequently, Nrf2 accumulates and induces transcription of ARE/EpRE-driven genes. We also show that in addition to activating Nrf2, Kyn-CKA inhibits the expression of genes encoding a panel of pro-inflammatory proteins in primary murine macrophages, in agreement with published work [21], and further reveal Nrf2, but not AhR, as one of the main contributors to the acute anti-inflammatory activity of Kyn-CKA in these cells.

## 2 Materials and methods

### 2.1 Materials

l-Kynurenine was purchased from Sigma (St. Louis, MO). CH-223191 (HY-12684) was from MedChemExpress LLC (Monmouth Junction, NJ), mouse M-CSF was from Prospec (Rehovot, Israel), Kyn-CKA [a mixture of two stereoisomers, (*E*)- and (*Z*)-(4-(2-aminophenyl)-4-oxobut-2-enoic acids; n.b., Fig. 3A shows only the structure of the *E* isomer] and Dean-CKA [(*E*)-4-oxo-4-phenylbut-2-enoic acid)] were custom-synthesized by LGC Standards (Manchester, NH). (±)-TBE-31 was synthesized as described previously [23]. All other chemicals were of analytical grade and obtained from Sigma unless specified. ELISA kits for MCP1 (DY479 and MJE00B) and IL6 (DY406) were obtained from R&D Systems (Minneapolis, MN). The AhR reporter assay kit (M06001) was obtained from INDIGO Biosciences (State College, PA). For this assay, l-kynurenine solutions were incubated with 35 mg of 3-mercaptopropyl-functionalized silica (SiliCycle, Quebec City, Canada) for 15 min at room temperature to remove potential Kyn-CKA traces, followed by 0.22-μm filtration.

### 2.2 Animals

Animal breeding and maintenance was in accordance with the regulations described in the UK Animals (Scientific Procedures) Act 1986, and with the approval by the Welfare and Ethical use of Animals Committee of the University of Dundee and the Institutional Animal Care and Use Committee of the University of Alabama at Birmingham (22775). Wild-type (WT), Nrf2-knockout (Nrf2-KO) and Keap1-knockdown (Keap1-KD) C57BL/6J mice [24] were bred and maintained at the WTB Resource Unit of the University of Dundee on a 12-h light/12-h dark cycle, 35 % humidity with free access to water and pelleted RM1 diet (SDS Ltd., Witham, Essex, UK). In Nrf2-KO mice and derived cells, Nrf2 is transcriptionally inactive due to disruption of the *Nfe2l2* locus by an in-frame insertion of the *lacZ* coding sequence, which replaces part of exon 4 and all of exon 5 [25]. This knock-in retains the Neh2 Keap1-binding domain of Nrf2, resulting in Nrf2 turnover similar to the wild-type protein, but lacks the CNC-bZIP DNA binding and dimerization domains of the transcription factor. In Keap1-KD mice and derived cells, loxP sites inserted into the *Keap1* locus yielded a hypomorphic ‘knockdown’ allele with reduced *Keap1* expression even without Cre, resulting in constitutive Nrf2 stabilization, increased nuclear Nrf2 levels, and elevated basal expression of Nrf2-target genes [26]. For the experiments shown in Fig. 7, Fig. 8, Fig. 9, Fig. 10, Nrf2-KO (B6.129X1-*Nfe2l2*^*tm1Ywk*^/J; RRID:IMSR_JAX:017009) and C57BL/6J (RRID:IMSR_JAX:000664) mice were purchased from the Jackson Laboratory. *LysM AhR*^*−/−*^ mice were generated in house by crossing LysM Cre (B6.129P2-Lyz2tm1(cre)Ifo/J; RRID:IMSR_JAX:004781) with *Ahr*^*flox/flox*^ mice (Ahrtm3.1Bra/J; RRID:IMSR_JAX:006203).

### 2.3 Cell culture

All cultured cells were maintained in a humidified atmosphere of 95 % air and 5 % CO_2_ at 37 °C and were routinely tested to ensure that they were mycoplasma-free. MEF cells isolated from mice expressing wild-type or mutant Keap1 [27] and U2OS cells stably expressing Keap1-mCherry or free mCherry [28] were cultured in Dulbecco's modified Eagle's medium (DMEM), supplemented with 10 % heat-inactivated FBS. ARPE-19 cells [28] were grown in DMEM, supplemented with 10 % heat- and charcoal-inactivated FBS.

**Fig. 1:**
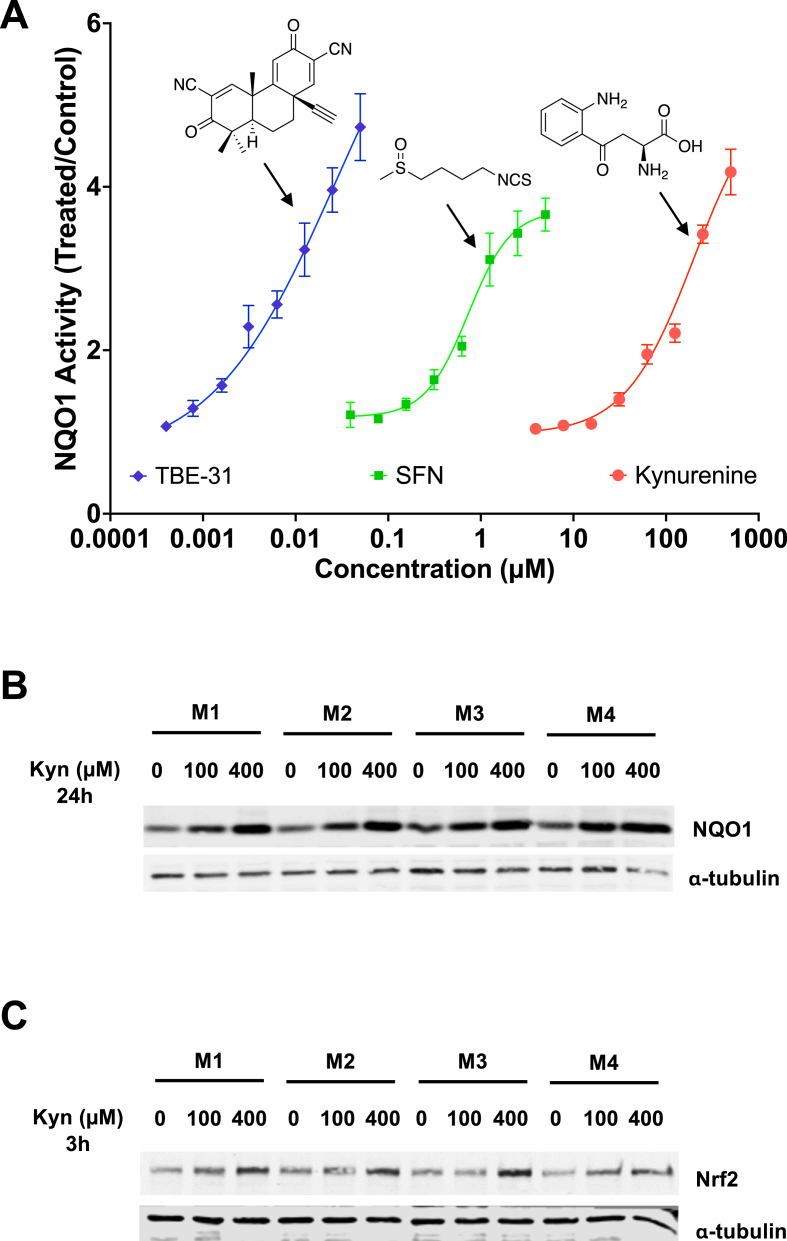
**Kynurenine is an Nrf2 activator in BMDMs. (A)** BMDMs were resuspended, seeded into 96-well plates (100,000 cells/well), and treated 24 h post-seeding with either vehicle [0.1 % DMSO (v/v)] or increasing concentrations of kynurenine, TBE-31, or sulforaphane (SFN). After 48 h, the specific enzyme activity of NQO1 was quantified in cell lysates. Data are shown as fold change in NQO1 specific enzyme activity relative to vehicle control. Values represent means of 8 biological replicates. Dose-response curves were fitted using a four-parameter logistic (4PL) model in GraphPad Prism. **(B,C)** BMDMs, derived from four WT C57BL/6J mice (M1-M4), were seeded into six-well plates (1 × 10^6^/well), allowed to adhere overnight, and subsequently treated with either vehicle or kynurenine (Kyn, 100 μM or 400 μM) for either 3 h or 24 h. Total cell lysates were prepared and the protein levels of NQO1 **(B)** were determined by immunoblotting 24 h post-treatment. The protein levels of Nrf2 **(C)** were determined by immunoblotting 3 h post-treatment. The levels of α-tubulin served as a loading control.

**Fig. 2:**
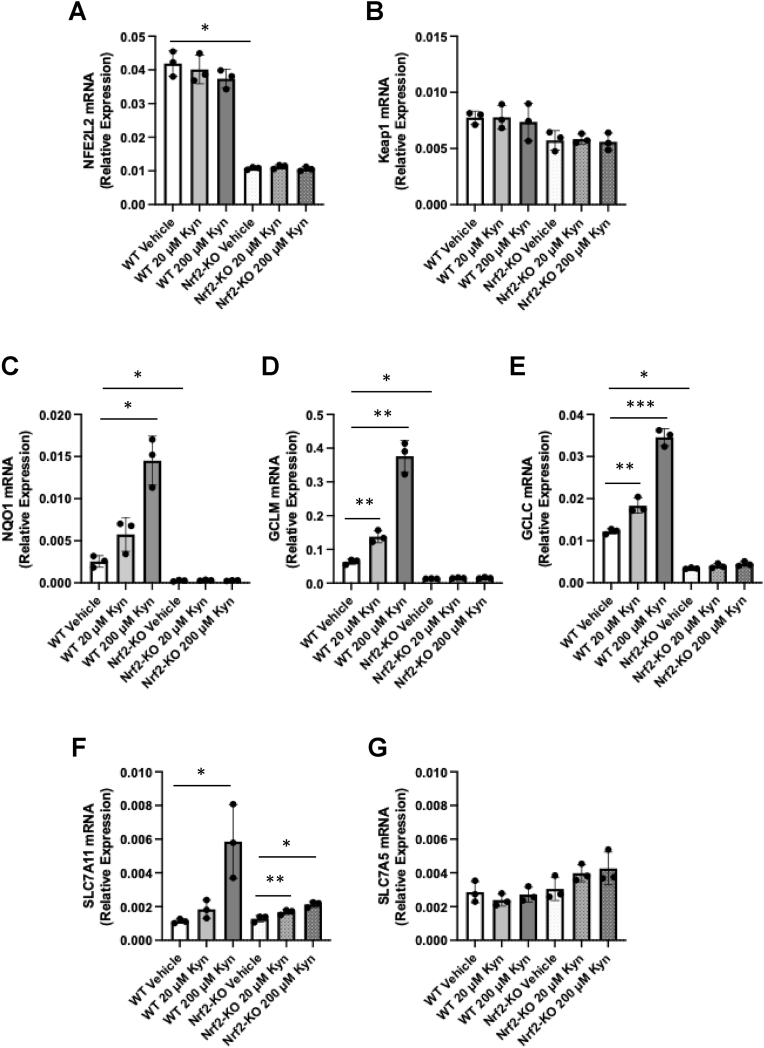
**Kynurenine induces Nrf2-driven transcription in BMDMs**. WT and Nrf2-KO BMDMs were seeded into six-well plates (1 × 10^6^/well), allowed to adhere overnight, and then treated for 18 h with vehicle or kynurenine (Kyn; 20 μM or 200 μM). The levels of mRNA for Nrf2 (gene name *Nfe2l2*) **(A)**, *Keap1***(B)**, and the Nrf2 transcriptional targets *Nqo1***(C)**, *Gclm***(D)**, *Gclc***(E)**, *Slc7a11***(F)**, and *Slc7a5***(G)** were determined by quantitative RT-PCR after normalization to the housekeeping control *Rps18* (or *B2m* for *Keap1*). Bars represent means ± SEM (n = 3 biologically independent replicates per condition), with individual data points shown. ∗, p < 0.05; ∗∗, p < 0.01; ∗∗∗, p < 0.001; ∗∗∗∗, p < 0.0001.

**Fig. 3:**
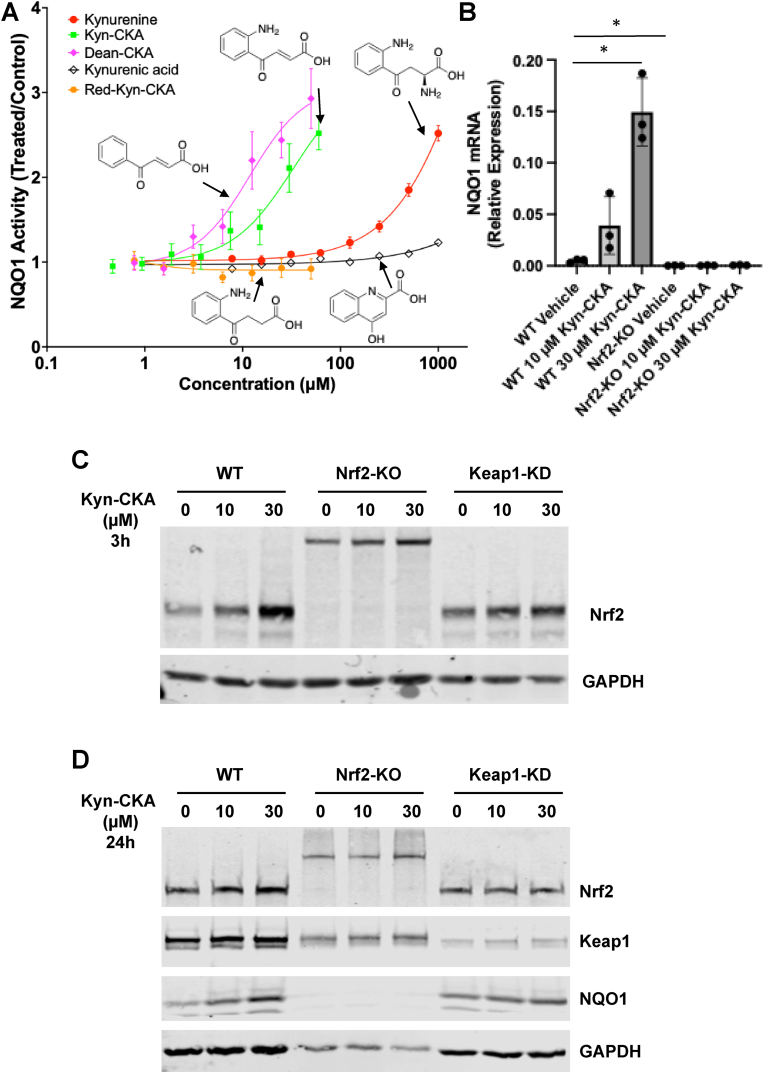
**Kynurenine-CKA is a Nrf2-dependent inducer of NQO1. (A)** Human ARPE-19 cells were seeded into 96-well plates (10,000 cells/well) and treated 24 h post-seeding with either vehicle [0.1 % MeOH (v/v)] or increasing concentrations of: kynurenine, Kyn-CKA, the deaminated Kyn-CKA derivative Dean-Kyn-CKA, the reduced Kyn-CKA derivative Red-Kyn-CKA, or kynurenic acid (KynA). After 24 h, the specific enzyme activity of NQO1 was quantified in cell lysates. Data are shown as fold change in NQO1 specific enzyme activity relative to vehicle control. Values represent means of 8 biological replicates. Dose-response curves were fitted using a four-parameter logistic (4 PL) model in GraphPad Prism. **(B)** WT and Nrf2-KO BMDMs were seeded into six-well plates (1 × 10^6^/well), allowed to rest overnight, and then treated for 18 h with vehicle or Kyn-CKA (10 μM or 30 μM). The levels of mRNA for *Nqo1* were determined by quantitative RT-PCR after normalization to the housekeeping control, *B2m*. Bars represent means ± SEM (n = 3 biologically independent replicates per condition), with individual data points shown. ∗, p < 0.05. **(C,D)** WT, Nrf2-KO and Keap1-KD BMDMs were seeded into six-well plates (1 × 10^6^/well), allowed to rest overnight, and subsequently treated with vehicle or Kyn-CKA (10 μM or 30 μM). Whole-cell lysates were collected after 3 h and 24 h and analysed by immunoblotting for Nrf2 at the 3 h timepoint **(C)** and for Nrf2, Keap1 and NQO1 at the 24h timepoint **(D)**, with GAPDH serving as a loading control.

Mouse BMDM cells were obtained by *in vitro* differentiation of precursor cells isolated by flushing the tibia and femur with high-glucose DMEM containing 10 % heat-inactivated FBS. Precursor cells were differentiated with either 20 % L929 medium (for the experiments shown in Fig. 1, Fig. 2, Fig. 3) or M-CSF (10 ng/mL, for the experiments shown in Fig. 7, Fig. 8, Fig. 9, Fig. 10) for 7 days in high-glucose DMEM, 10 % heat-inactivated FBS and 1 % penicillin/streptomycin. For the experiments shown in Fig. 1, Fig. 2, Fig. 3, on day 7, adherent cells were detached with 5 mM EDTA in PBS (10 min, 37 °C), counted and replated in complete media, at the cell densities indicated in the legends. For the experiments shown in Fig. 7, Fig. 8, Fig. 9, Fig. 10, cells were seeded in 12-well plates (0.5 × 10^6^/well) in complete media and incubated at 37 °C, 5 % CO_2_. Experimental treatments were performed in the absence of FBS for the indicated durations. For LPS treatments (*E. coli* O111:B4), cells were pre-treated with Kyn-CKA for 40 min prior to endotoxin addition.

### 2.4 NQO1 enzyme activity assay

NQO1 inducer activity was determined using the “Prochaska” microtiter plate bioassay [29]. Cells were grown in 96-well plates for 24 h, and then exposed in 8 replicates to 2-fold serial dilutions of each compound or vehicle for a further 24 h or 48 h, as indicated in the figure legends. After washing with PBS (200 μL, twice), cell lysates were obtained by adding 75 μL digitonin (0.8 g/L in 2 mmol/L EDTA, pH 7.8) followed by incubation at room temperature for 10 min with shaking. Aliquots of cell lysates (20 μL) were transferred to parallel 96-well plates, and protein concentrations were measured by the bicinchoninic acid (BCA) assay (Thermo Fisher). The enzyme activity of NQO1 (with menadione as a substrate) was quantified using the remaining 55 μL of lysate. Inducer potency was expressed as a CD value, i.e., the Concentration that Doubles the NQO1 specific enzyme activity.

### 2.5 Immunoblotting

Following treatment, cells were washed twice with ice-cold PBS and lyzed in SDS lysis buffer [50 mM Tris pH 6.8, 10 % glycerol (v/v), 2 % SDS (w/v)]. The lysates were sonicated for 30 s (using 5 s on - 1 s off cycles) at 20 % amplitude, the protein concentrations were determined using the BCA assay (Thermo Fisher). Samples were adjusted to the same protein concentration, reduced by adding 2-mercaptoethanol to a final concentration of 5 % (v/v), incubated for 30 min at room temperature and then heated at 95 °C for 5 min before being subjected to electrophoresis. Bromophenol Blue [0.001 % (w/v)] was added, and equal sample volumes containing equal amounts of total protein, were loaded onto either Tris-Glycine or 4–12 % Bis-Tris NuPAGE gels (Thermo Fisher). Proteins were separated by electrophoresis using either Tris-Glycine or MOPS (Thermo Fisher) buffer and transferred onto 0.45-μm premium nitrocellulose membranes (Amersham) by wet transfer (Bio-Rad). Transfer efficiency was confirmed by membrane incubation with 0.1 % (w/v) Ponceau S solution in 5 % (v/v) acetic acid. Stain was then washed off by PBS-0.1 % (v/v) Tween 20 (PBST), after which membranes were blocked in 5 % (w/v) non-fat milk (Marvel) dissolved in PBST (milk-PBST) for 1 h at room temperature on an orbital shaker. The membranes were incubated with primary antibodies overnight at 4 °C or for 1 h at room temperature for loading controls (e.g. GAPDH, α-tubulin or β-actin), washed with PBST (3 times, 10 min each), and incubated with fluorescently conjugated secondary antibodies (1:20000) (LI-COR) for 1 h at room temperature. The blots were then washed with PBST (3 times, 10 min each) at room temperature, and the proteins were visualized using the Odyssey CLx imager (LI-COR). The primary antibodies and their respective dilutions and sources were: Keap1 (1:4000, clone 144, Millipore MABS514); NQO1 (1:1000, clone D6H3A, Cell Signaling 62262); Nrf2 (1:1000, clone E5F1A, Cell Signaling 20733); GAPDH (1:30000, clone 1E6D9, Proteintech 60004-1-Ig); β-actin (1:10000, clone AC-15, Sigma-Aldrich A5441); and α-tubulin (1:10000, clone DM1A, Cell Signaling 3873). All antibodies were diluted in milk-PBST.

### 2.6 mRNA analysis

Cells were lysed and total RNA extracted either using PureLink™ RNA Mini Kit (Thermo Fisher) or TRIzol reagent (Invitrogen, USA; 0.4 mL per million cells) and quantified by UV spectroscopy at 260 nm or NanoDrop™ One/OneC. Reverse transcription was performed using either PrimeScript™ RT Master Mix kit (Takara Bio) or iScript cDNA kit (Bio-Rad). Real-time PCR was carried out on Applied Biosystems QuantStudio™ 5 or 7 Real-Time PCR systems using TaqMan Fast Advanced Master Mix (Thermo Fisher) or PowerUp™ SYBR™ Green Master Mix (Thermo Fisher). Results were calculated using the ΔΔCt method and normalized to the corresponding housekeeping gene(s) selected for each experiment. The TaqMan™ Gene Expression Assay IDs (Thermo Fisher, Waltham, MA) were: Mm01253561 (for *Nqo1*); Mm00516005 (for Ho1); Mm01324400 (for *Gclm*); Mm00434228 (for *IL1b*); Mm00446190 (for *IL6*); Mm00441242 (for *Mcp1*); Mm00443252 (for *Tnf*); Mm004405021 (for *Nos2*); Mm00437762 (for *B2m*); Mm03024075 (for *Hprt1*); and 4352341E (for *Actb*). The sequences of the primers for the SYBR™ Green Real-Time PCR were:

**Table:** 

*B2m *(Mm)	F CCTGTATGCTATCCAGAAAACCCCTC
	R ATCACATGTCTCGATCCCAGTAGACG
*Gapdh *(Mm)	F ATCACTGCCACCCAGAAGACTG
	R CATCATACTTGGCAGGTTTCTCC
*Gclc* (Mm)	F TGGACACCCGATGCAGTATTC
	R ATCCACCTGGCAACAGTCATTAG
*Gclm* (Mm)	F CACCAGATTTGACTGCCTTTGCTA
	R CCAATCCTGGGCTTCAATG
*Keap1 *(Mm)	F ATTGAGTTCGCCTACACGGCCTC
	R TGGCAGTGTGACAGGTTGAAGAACTC
*Nfe2l2* (Mm)	F AGCTACTCCCAGGTTGCCCACATTCC
	R CAGGGCAAGCGACTCATGGTCATC
*Nqo1 *(Mm)	F GCTGCAGACCTGGTGATATT
	R ACTCTCTCAAACCAGCCTTT
*Rps18 *(Mm)	F GGATGTGAAGGATGGGAAGT
	R CCCTCTATGGGCTCGAATTT
*Slc7a5 *(Mm)	F CTTCGGCTCTGTCAATGGGT
	R TTCACCTTGATGGGACGCTC
*Slc7a11 *(Mm)	F CTTTGTTGCCCTCTCCTGCTTC
	R CAGAGGAGTGTGCTTGTGGACA

### 2.7 Kyn-CKA reactions with N-acetyl cysteine or Keap1-BTB-C151

The WT Keap1 BTB domain protein and the Keap1-BTB-C151S mutant protein were expressed and purified as previously described [9]. The proteins were reduced with 1 mM DTT at room temperature for 1 h and subsequently buffer exchanged into degassed 100 mM phosphate buffer pH 7.5 using Zeba 7 kDa MWCO spin desalting columns. To avoid rapid oxidation of C151, the protein was used within 30 min or stored under argon. Kyn-CKA was preincubated in 100 mM phosphate buffer (pH 7.5) for 2 h then reacted with NAC, WT Keap1-BTB, or Keap1-BTB-C151S (or buffer control) in the same buffer. Reactions were initiated by adding Kyn-CKA to cuvettes containing NAC, WT Keap1-BTB, Keap1-BTB-C151S, or buffer as a control. Final concentrations were 30 μM Kyn-CKA and 30 μM Keap1-BTB-C151 or Keap1-BTB-C151S. Given the C151 p*K*_a_ of 6.9 [9], 24 μM C151 thiolate is available to react at pH 7.5. Correspondingly, to obtain a final reaction concentration of 24 μM thiolate NAC, with a p*K*_a_ of 9.4, the final concentration of NAC was 2.5 mM in the reactions. Absorbance spectra were collected on an Agilent Cary 60 UV–Vis spectrophotomer. While NAC alone contributes no absorbance, and thus the reaction is monitored simply as the change in the Kyn-CKA absorbance peak, the Keap1-BTB protein alone does contribute absorbance. Thus, the Keap1-BTB spectrum and the Kyn-CKA spectrum were mathematically added to demonstrate the expected peak of their mixture, pre-reaction. To determine the wavelength at which absorbance has the greatest change due to the reaction, this added spectrum was subtracted from the reaction of Kyn-CKA with Keap1-BTB at equilibrium (i.e., post-reaction minus pre-reaction), thus, the kinetics of the reaction were monitored at 410 nm. To account for baseline differences between runs, we took advantage of the fact that all reactions were monitored until equilibrium was achieved. The last four absorbance values of a given run, at equilibrium, were averaged, and subtracted from the absorbance at each timepoint for a given run. After plotting the ΔAbs_410 nm_ versus time, data showing a change over time were fitted to a one-phase decay.

### 2.8 Mass spectrometry analyses

Whole BTB Protein Mass Analysis: For analysis of the products of the reaction between the WT Keap1-BTB and Kyn-CKA, whole protein sample was loaded onto a Phenomenex 2.1 × 100 mm, 1.6 μm Luna Omega, 80 Å reverse-phase column (Torrance, CA). Analytes were eluted using a linear gradient of 5–50 % mobile phase B for 10 min and 50–98 % B until 11 min with a 1-min hold, followed by re-equilibration at initial conditions for 8 min using an Exion UHPLC (Sciex, Toronto, Ontario) and a flow rate of 500 μL/min. The mobile phases were A) ddH2O with 0.1 % formic acid and B) acetonitrile with 0.1 % formic acid respectively. A SCIEX 7600+ ZenoTOF mass spectrometer (SCIEX, Toronto, Canada) was used to analyze the protein charge state profile in the positive ion mode. The IonSpray voltage was +5500 V and the declustering potential was 80 V. GS1/GS2 and curtain gases were set at 70/30 and 35 psi, respectively. The interface heater temperature was 500 °C. Eluted protein(s) were subjected to a 100 ms time-of-flight survey scan from 400 to 3000 *m*/*z*. The whole protein spectra were centroided and exported as peak list text files and uploaded to UniDec Software 6.01 (https://unidec.chem.ox.ac.uk/) for whole mass calculations.

Keap1-BTB Adduct Identification: An aliquot of Kyn-CKA modified Keap1-BTB protein (∼30 μM) was dissolved in 8 M urea and alkylated with 55 mM Iodoacetamide for 30 min at room temperature. The solution was then diluted 10-fold with 100 mM ammonium bicarbonate (pH 8.5) and digested overnight with Promega Platinum mass spectrometry grade trypsin (Madison, WI) at 37 °C. The digestion was then quenched with 100 mM HCl and followed by mass spectrometry analysis. Samples were loaded onto a Phenomenex 2.1 × 100 mm, 1.6 μm Luna Omega, 80 Å reverse-phase column (Torrance, CA). A linear gradient was used consisting of 2–50 % mobile phase B for 36 min and 50–98 % B until 44 min with a 4-min hold, followed by re-equilibration at the initial conditions for 12 min using an Exion UHPLC and a flow rate of 250 μl/min. The mobile phases were A) ddH_2_O with 0.1 % formic acid and B) acetonitrile with 0.1 % formic acid, respectively. A SCIEX 7600+ ZenoTOF mass spectrometer was used to analyze the peptide profile in the positive ion mode. The IonSpray voltage was +5500 V, the declustering potential was 80 V, and GS1/GS2 and curtain gases were set at 70, 30 and 35 psi, respectively. The interface heater temperature was 500 °C. Eluted peptides were subjected to a 100-ms time-of-flight survey scan from 400 to 1250 *m*/*z* to determine the top twenty most intense ions for MSMS analysis. Product ion time-of-flight scans at 25 msec were carried out to obtain the tandem mass spectra of the selected parent ions over the range from *m*/*z* 100–2000. Spectra were centroided and de-isotoped by Analyst software OS Software 3.4 (Sciex, Toronto, Ontario). The tandem mass spectrometry data were processed to provide protein identifications using an in-house Protein Pilot 5.02 search engine (SCIEX, Toronto, Canada) utilizing trypsin digestion parameter profile. Peptides with modified cysteines with Kyn-CKA were *de novo* sequenced and interpreted using PeakView 2.2 software (SCIEX, Toronto, Canada).

NAC-Kyn-CKA Characterization: Aliquots of the reaction between NAC and Kyn-CKA (0.5 pmoles adduct) were loaded onto a Luna Omega 2.1 × 100mm 1.6-μm column (Phenomenex) at a flow rate of 0.35 mL/min using the following solvent system: A) ddH_2_O + 0.1 % formic acid, B) acetonitrile + 0.1 % formic acid. Samples were loaded at 1 % B and eluted with a linear gradient from 1 to 70 % of B over 7 min. The column was then washed with 100 % B for 2 min and re-equilibrated at 1 % B for 4 min. Fragmentation analysis was performed using a 7500+ Triple Quadruple Mass Spectrometer (Sciex) in the positive ion mode using curtain gas: 40 psi, ion spray voltage: +5500 V, temperature: 550 °C, ion source gas 1: 65 psi, ion source gas 2: 50 psi, collision gas: 6. Ions were selected between *m*/*z* 300–400 and fragmented using the following parameters: Entrance potential (EP): 6 V; Collision cell exit potential (CXP): 12 V, and Collision Energy (CE): 20 V. For multiple reaction monitoring analyses, the following transitions were used to identify the NAC-Kyn-CKA adduct in the positive ion mode: 355.1/192.0 (CE: 17 V) and 355.1/174.0 (CE: 20 V).

### 2.9 Cellular Thermal Shift Assay (CETSA)

CETSA was performed as described [28,30]. U2OS cells stably expressing Keap1-mCherry or mCherry were induced with doxycycline hyclate (0.5 μg/mL) for 48h. Cells were washed three times with ice-cold PBS and then collected by scraping into 1.0 mL PBS supplemented with protease inhibitor cocktail (Roche). Cell suspensions were snap-frozen in liquid nitrogen and stored at −70 °C. Subsequently, cells were lyzed by six freeze-thaw cycles, and cell debris was removed by centrifugation at 9600×*g* at room temperature for 5 min. Clarified cell lysates were collected, and protein concentrations were measured using the BCA assay (Thermo Fisher). Samples were normalized, ensuring control and treated groups were adjusted to the same protein concentration, and then incubated with either Kyn-CKA or vehicle [0.1 % MeOH (v/v)] for 1 h at 37 °C. The incubation mixtures were aliquoted (100 μL) and heated for 3 min at the indicated temperatures (i.e. from 37 °C to 70 °C, in 3 °C increments), and the aggregated material was removed by centrifugation at 17,000×*g* at 4 °C for 40 min. The fluorescence in the soluble fraction was measured with 580 nm excitation, 590 nm cut-off and 615 nm emission wavelengths.

For ITDRF-CETSA, cell lysates were aliquoted (100 μL) and incubated with a range of compound concentrations or vehicle [0.9 % MeOH (v/v)] for 1 h at 37 °C to allow compound-target interaction. Following incubation, samples were subjected to heat treatment at 64 °C for 3 min. The aggregated material was removed by centrifugation and the fluorescence in the soluble fraction was measured as described above.

### 2.10 AhR reporter assay

The assay was performed following the manufacturer's instructions. Briefly, 200 μL of mouse AhR reporter cells expressing luciferase were seeded in 96-well plates and incubated at 37 °C and 5 % CO_2_. After 6 h, the culture media was replaced with treatment media. Reporter cells were treated with variable concentrations of L-Kynurenine, Kyn-CKA or Dean-Kyn-CKA (0–30 μM) for 24 h. Alternatively, they were pre-incubated with 10 μM CH223191 (AhR inhibitor) for 1 h, followed by treatment with 10 μM L-Kynurenine or Kyn-CKA, in the presence of 10 μM AhR inhibitor. After 24 h, the media were discarded, and Luciferase Detection Reagent was added followed by luminescence measurement using a Spectramax iD5 (Molecular Devices) plate reader.

### 2.11 Quantification and statistical analysis

All quantitative data are represented graphically as mean values ± 1 standard deviation (SD) or standard error of means (SEM), as indicated in the figure legends. Student's t-test (for two-group comparisons) or one-way ANOVA (for multiple comparisons) and either Tukey's or Dunnett's post-test (indicated in the figure legends) were used to test for statistical significance.

## 3 Results

### 3.1 Kynurenine induces Nrf2-driven transcription in murine bone marrow-derived macrophages

Our previous high-resolution proteomic analysis showed that the Keap1/Nrf2 system is fully functional in primary murine bone marrow-derived macrophages (BMDMs), and its genetic or pharmacologic activation suppresses their pro-inflammatory responses to lipopolysaccharide (LPS) whilst supporting their redox metabolism and mitochondrial adaptation to inflammatory stress [31]. Thus, we employed this system to explore the possibility that kynurenine activates Nrf2 in a physiologically relevant cellular model using a quantitative bioassay that measures the enzyme activity of the classical Nrf2 target NQO1 [32]. Treatment with kynurenine for 48 h led to a concentration-dependent increase in NQO1 specific enzyme activity (Fig. 1A). However, the concentration required to double the NQO1 enzyme activity (CD value) was higher (CD = 100 μM) compared to two other well-established NQO1 inducers, the tricyclic cyanoenone TBE-31 (CD = 0.03 μM) and the isothiocyanate sulforaphane (SFN, CD = 0.6 μM), indicating that kynurenine is a relatively low potency NQO1 inducer. The concentration-dependent induction of NQO1 by kynurenine was further confirmed by immunoblotting of lysates of cells that had been treated with kynurenine for 24 h (Fig. 1B); this type of analysis also showed that the 3 h-kynurenine treatment increased the abundance of Nrf2 protein (Fig. 1C).

To examine whether the kynurenine-mediated upregulation of NQO1 (and other Nrf2-transcriptional targets) is dependent on Nrf2, RT-qPCR analysis was performed on BMDMs isolated from wild-type (WT) or *Nfe2l2*^−/−^ (i.e., Nrf2-KO) mice. In cells from Nrf2-KO mice, Nrf2 is transcriptionally inactive [25]. BMDMs of the two genotypes were treated with vehicle, 20 μM, or 200 μM kynurenine for 18 h, and the mRNA levels for *Nfe2l2*, *Keap1*, and the Nrf2 target genes *Nqo1*, *Gclc*, *Gclm*, *Cd36* and *Slc7a11* were quantified. Notably, the *Nfe2l2* mRNA levels were substantially lower in vehicle-treated Nrf2-KO BMDMs (Fig. 2A). Although to a much smaller extent, the *Keap1* mRNA levels were also lower in vehicle-treated Nrf2-KO BMDMs in comparison with their WT counterparts (Fig. 2B).

In WT BMDMs, kynurenine caused a robust, concentration-dependent increase in the mRNA levels of the Nrf2-target genes, with *Nqo1* and *Gclm* showing greater than five-fold upregulation in cells treated with 200 μM kynurenine (Fig. 2C–E). In contrast, in Nrf2-KO BMDMs, the basal and the inducible expression of *Nqo1*, *Gclc* and *Gclm* was strongly suppressed, or absent, demonstrating clear Nrf2 dependence. One exception was *Slc7a11* (Fig. 2F)*,* whose basal levels were similar between WT and Nrf2-KO cells, and its expression remained partially inducible in Nrf2-KO BMDMs, albeit to a much smaller (1.7-fold in Nrf2-KO vs. 5.2-fold in WT BMDMs treated with 200 μM kynurenine) magnitude. This result suggests that the expression of *Slc7a11* involves additional transcriptional inputs, such as ATF4, which has also been shown to be activated by kynurenine [33]. Importantly, the expression of *Slc7a5* was unaffected by neither genotype nor kynurenine treatment (Fig. 2G), indicating that the transport capacity of large neutral amino acids (including tryptophan and kynurenine) is Nrf2-independent in this system. Together with previously published work [21,33], these results establish that the endogenous metabolite kynurenine is an inducer of a broad transcriptional program in macrophages via a mechanism that is dependent on Nrf2.

### 3.2 Kynurenine-CKA is a Nrf2-dependent inducer of NQO1

High levels of kynurenine in cells, and *in vivo*, lead to the generation of the electrophilic metabolite kynurenine-carboxyketoalkene (Kyn-CKA), through spontaneous deamination [21]. Unlike kynurenine, which is not electrophilic, Kyn-CKA contains an α,β-unsaturated carbonyl, a Michael acceptor moiety, which is a characteristic feature of many compounds that inhibit Keap1 by reacting with C151, block ongoing proteasomal degradation of Nrf2, and induce NQO1 activity [34,35]. Treatment with Kyn-CKA was previously shown to induce Nrf2-dependent gene expression in mice *in vivo* as well as in the murine hepatocyte cell line AML-12 and MEF cells derived from wild-type, but not Nrf2-knockout mice [21]. These data, together with the electrophilic properties of Kyn-CKA and the high concentrations of kynurenine that were required to induce NQO1, led us to hypothesize that Kyn-CKA, and not kynurenine itself, was the actual inducer.

To test this hypothesis, we performed a small structure-activity relation study comparing the NQO1 inducer activity of kynurenine and Kyn-CKA, in which we also included the following: (i) Dean-Kyn-CKA, the deaminated derivative of Kyn-CKA, which lacks the *ortho*-aminophenyl group (NH_2_), and so prevents intramolecular cyclisation that would otherwise lead to loss of electrophilicity; (ii) Red-Kyn-CKA, a non-electrophilic form of Kyn-CKA in which the α,β-unsaturated carbonyl is reduced; and (iii) kynurenic acid (KynA), an endogenous biologically active non-electrophilic metabolite of kynurenine. For this comparison, we used the human ARPE-19 cell line, which we have previously shown to respond robustly to compounds that increase Nrf2 activity [36]. Both Kyn-CKA and its deaminated derivative (Dean-Kyn-CKA) produced a clear concentration-dependent induction of NQO1 (Fig. 3A), with Dean-Kyn-CKA displaying higher potency (CD = 10 μM) than Kyn-CKA (CD = 30 μM). As expected, kynurenine also induced NQO1 in a concentration-dependent manner, but with much lower potency (CD = 400 μM). KynA elicited only a very slight (1.2-fold) increase in NQO1 activity at the highest concentrations tested, and the reduced, non-electrophilic analog (Red-Kyn-CKA) was inactive. Together, these data define a clear structure-activity relationship where the electrophilicity of Kyn-CKA is essential for robust NQO1 induction, and also further strengthen the notion that Kyn-CKA rather than kynurenine itself, is the actual inducer. In agreement with these results in ARPE-19 cells, Kyn-CKA was also much more potent than kynurenine in inducing NQO1 in BMDMs: the NQO1 mRNA level was increased by 30-fold after treatment with 30 μM Kyn-CKA (Fig. 3B), whereas 5.7-fold induction was achieved by 200 μM kynurenine (Fig. 2C).

To determine whether, similar to its kynurenine precursor, the ability of Kyn-CKA to induce NQO1 is Nrf2-dependent, BMDMs were derived from WT, Nrf2-KO and Keap1-KD mice. In unstimulated WT cells, immunoblotting showed a robust, concentration-dependent increase in Nrf2 protein levels 3 h post-treatment with Kyn-CKA (Fig. 3C), and induction of NQO1 at the protein level at the 24 h timepoint (Fig. 3D). In contrast, both the mRNA (Fig. 3B) and the protein (Fig. 3D) NQO1 levels were low, and its induction was completely abolished in Nrf2-KO BMDMs. Notably, at the 3 h timepoint, the levels of the transcriptionally inactive Nrf2-lacZ fusion protein were increased by Kyn-CKA in the Nrf2-KO BMDMs, suggesting its turnover by Keap1. Stabilization of this fusion protein by Kyn-CKA is in agreement with previous reports that examined classical Nrf2 activators, e.g., sulforaphane in primary cultures of astrocytes obtained from this Nrf2-KO mouse strain [37]. Consistent with de-repression of Nrf2, Keap1-KD BMDMs exhibited elevated basal levels of both Nrf2 and NQO1, and Kyn-CKA treatment led to very mild induction. The protein levels of Keap1 remained largely unchanged by the Kyn-CKA treatment in each genotype (Fig. 3D), suggesting that pathway activation occurs via post-translational modulation of Nrf2 stability rather than altered Keap1 abundance.

### 3.3 Kyn-CKA increases the thermostability of Keap1

**Fig. 4:**
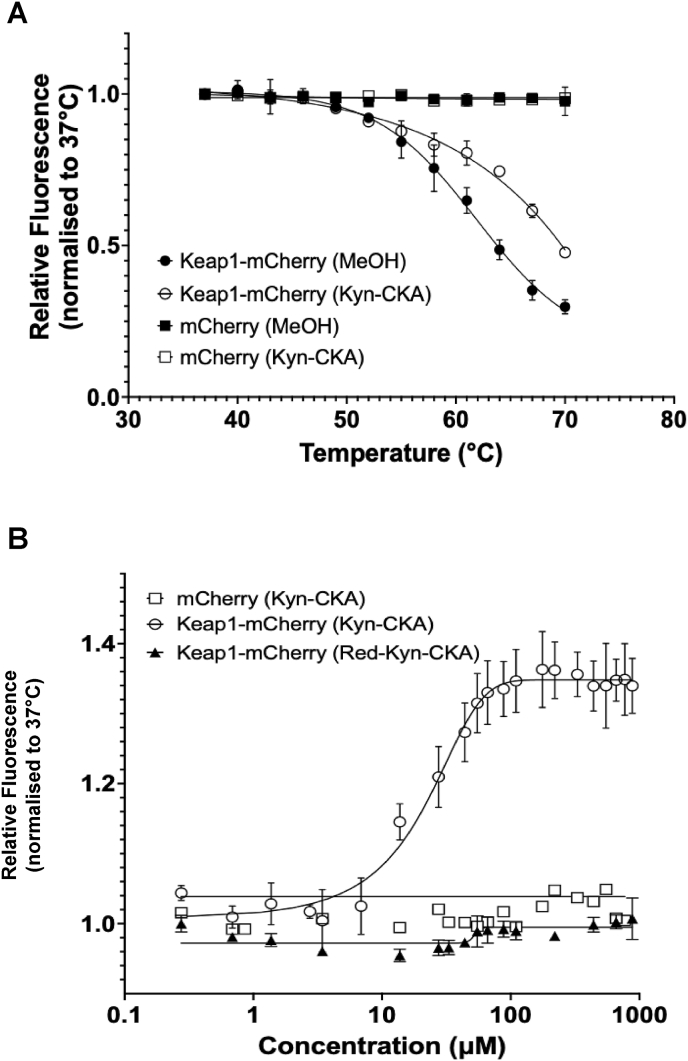
**Kyn-CKA increases the thermostability of Keap1-mCherry. (A)** Temperature-induced aggregation curves of Keap1-mCherry (n = 5) and mCherry (n = 3) following a 1 h incubation of lysates from Keap1-mCherry- or free mCherry-expressing U2OS cells with 110 μM Kyn-CKA at 37 °C. Curve fitting was performed using a four-parameter logistic (4 PL) model in GraphPad Prism. **(B)** Isothermal dose-response fingerprint (ITDRF) CETSA showing concentration-dependent stabilization of Keap1-mCherry by Kyn-CKA. Lysates of cells expressing Keap1-mCherry (n = 3) or free mCherry (n = 1) were incubated with Kyn-CKA for 1 h at 37 °C. Lysates of Keap1-mCherry-expressing cells (n = 3) were also incubated with the reduced analogue Red-Kyn-CKA. After compound incubation, cells were heated at 64 °C, aggregated proteins were removed by centrifugation, and the mCherry fluorescence was measured in the soluble fraction. Data are means ± SEM. A four-parameter logistic (4 PL) fit (X is log of concentration) is shown.

In addition to its substrate adaptor function, which leads to Nrf2 repression, Keap1 also serves as a cellular sensor for electrophiles [38]. To investigate whether Kyn-CKA engages Keap1, we employed the cellular thermal shift assay (CETSA), a widely used method for detecting changes in the thermal stability of protein targets in response to ligand binding or conformational alterations [39]. Incubation for 1 h at 37 °C of Kyn-CKA with lysates of U2OS cells stably expressing doxycycline (Dox)-inducible Keap1-mCherry fusion protein, or free mCherry as a control [28,30], increased the thermal stability of Keap1-mCherry, but not of free mCherry (Fig. 4A), indicating that Kyn-CKA binds the Keap1 portion of the fusion protein. Next, the isothermal dose-response fingerprint CETSA (ITDRF^CETSA^) [40] was employed to quantify compound-target engagement by measuring the thermal stability of Keap1-mCherry at 64 °C (the temperature yielding the greatest difference in thermal stability between the vehicle control and Kyn-CKA treatments) following incubation of cell lysates with Kyn-CKA, at a range of concentrations, for 1 h at 37 °C. A sigmoidal curve was fitted to the data, from which an apparent IC_50_ value of 12 μM was calculated, representing the concentration of Kyn-CKA required for half-maximal thermal stabilization of Keap1-mCherry (Fig. 4B). As expected, Kyn-CKA did not alter the stability of free mCherry protein at any of the concentrations tested. Crucially, an identical ITDRF^CETSA^ experiment with Red-Kyn-CKA instead of Kyn-CKA failed to affect the thermostability of Keap1-mCherry, further confirming the importance of electrophilicity of Kyn-CKA for engaging Keap1.

### 3.4 C151 in Keap1 is required for the activation of Nrf2 by Kyn-CKA

Kyn-CKA forms covalent adducts with the reactive thiol groups of both glutathione and free cysteine [21]. Together with the observed increase in the thermostability of Keap1, this strongly suggests that Kyn-CKA can similarly modify sensor cysteine(s) within Keap1. Such covalent modification(s) would impair the substrate adaptor function of Keap1 to target Nrf2 for ubiquitination and degradation, leading to stabilization of the transcription factor, its accumulation in the nucleus, and increased expression of Nrf2-target genes, as observed in the previous experiments (Fig. 3) . Although the CETSA experiment indicated engagement of Keap1 upon treatment with Kyn-CKA, it was possible that the observed thermostabilization of Keap1 was a consequence of non-specific conformational changes rather than covalent modification(s). Moreover, this experiment did not establish the identity of the sensor cysteine(s) within Keap1. To determine the main cysteine sensor(s) of Keap1 that are required for the activation of Nrf2 by Kyn-CKA, we used MEF cells derived from mice expressing either wild-type (WT) Keap1 or various mutant forms of Keap1, i.e. C151S, C226S, C613S, or C288E, and the double mutant C226S/C613S [27,41]. To establish that WT MEFs respond appropriately to electrophilic stimuli comparable to the other cell models used in the study, the NQO1 enzyme activity bioassay was performed following treatment with kynurenine, Kyn-CKA, Dean-Kyn-CKA, and Red-Kyn-CKA.

**Fig. 5:**
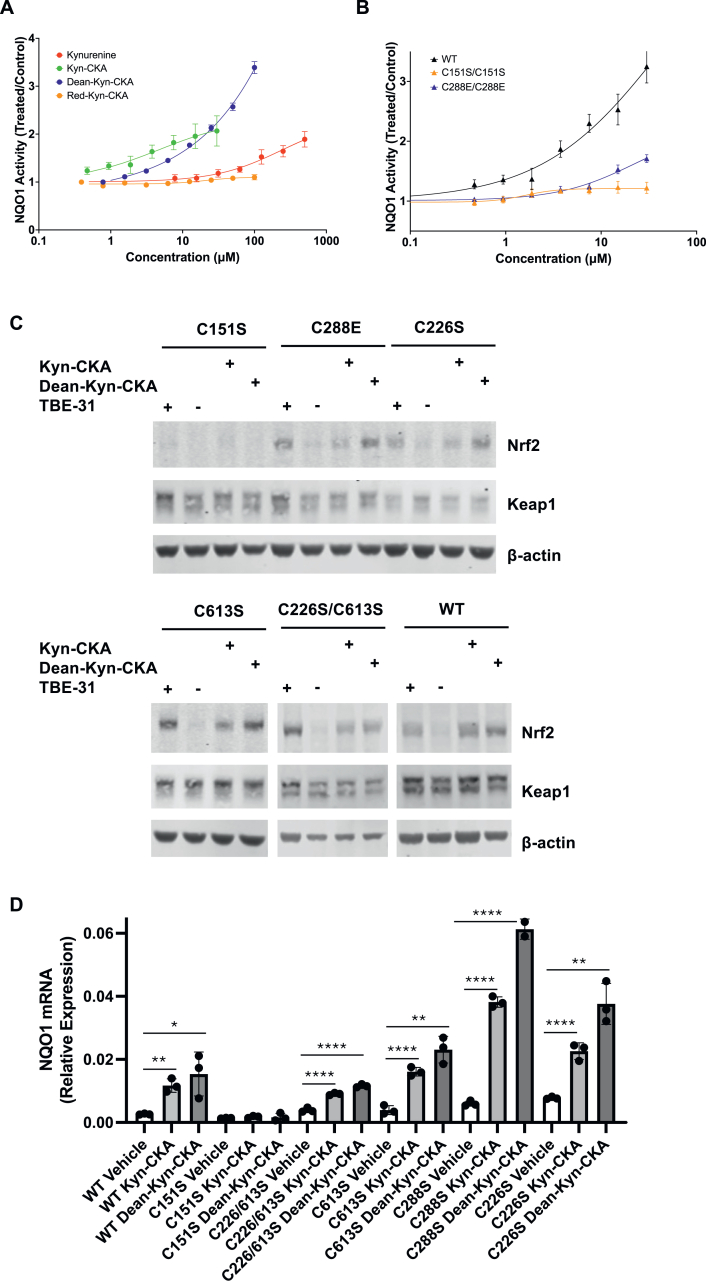
**C151 in Keap1 is the primary sensor for Kyn-CKA. (A)** MEF cells expressing wild-type Keap1 were seeded into 96-well plates (20,000 cells/well), and treated 24 h post-seeding with either vehicle [0.1 % MeOH (v/v)] or increasing concentrations of kynurenine, Kyn-CKA, the deaminated Kyn-CKA derivative Dean-Kyn-CKA, or the reduced Kyn-CKA derivative Red-Kyn-CKA. After 24 h, the specific enzyme activity of NQO1 was quantified in cell lysates. Data are shown as fold change in NQO1 specific enzyme activity relative to vehicle control. Values represent means of 8 biological replicates. Dose-response curves were fitted using a four-parameter logistic (4 PL) model in GraphPad Prism. **(B)** NQO1 enzyme activity measured using the same experimental conditions in MEF cells expressing either wild-type Keap1 (WT) or the cysteine mutants of Keap1 indicated in figure. **(C)** WT or Keap1 mutant MEFs were treated for 3 h with 10 nM TBE-31 (a known Nrf2 activator via C151 in Keap1 used as a control), vehicle [0.1 % MeOH (v/v)], 10 μM Kyn-CKA or 10 μM Dean-Kyn-CKA. Whole-cell lysates were collected and analysed by immunoblotting for Nrf2 and Keap1. β-actin served as a loading control. **(D)** Wild-type (WT) and Keap1 mutant MEFs (C151S/C151S, C288E/C288E, C226S/C226S, C226S/C613S and C613S/C613S) were treated with vehicle [0.1 % MeOH (v/v)], 10 μM Kyn-CKA or 10 μM Dean-Kyn-CKA for 18 h. The mRNA levels for *Nqo1* were quantified by RT-qPCR after normalization to the housekeeping control, *Gapdh*. Bars show means ± SEM; points denote biological replicates. ∗, p < 0.05; ∗∗, p < 0.01; ∗∗∗, p < 0.001; ∗∗∗∗, p < 0.0001.

In close agreement with the results in human ARPE-19 cells (Fig. 3A), both Kyn-CKA (CD = 30 μM) and Dean-Kyn-CKA (CD = 12.5 μM) induced a concentration-dependent increase in NQO1 activity in WT MEFs, whereas kynurenine was a much weaker inducer, and Red-Kyn-CKA was essentially inactive (Fig. 5A). Compared to WT cells, the mutant MEFs showed diminished inducer potency of Kyn-CKA, particularly in the C151S-Keap1 mutant MEFs (Fig. 5B), indicating C151 in Keap1 may be the primary mediator of Nrf2 activation. This conclusion was further supported by immunoblotting, which showed that treatment with 10 μM Kyn-CKA, 10 μM Dean-Kyn-CKA, or 10 nM TBE-31 (used as a positive control) increased the levels of Nrf2 in WT and all Keap1-mutant cells, except for MEFs harbouring C151S mutant Keap1 (Fig. 5C). Concordantly, the mRNA for NQO1 was robustly induced in WT and all mutant MEFs except for the C151S-Keap1 mutant cells (Fig. 5D), mirroring the enzyme activity data (Fig. 5A). Interestingly, the basal *Nqo1* mRNA levels were lower in the C151S-Keap1 mutant MEFs, suggesting that this cysteine also contributes to the expression of *Nqo1* under homeostatic conditions, perhaps due to its modification by endogenously produced electrophiles during cell metabolism [16], such as the glycolytic byproduct methylglyoxal, which has been shown to engage C151 [42].

**Fig. 6:**
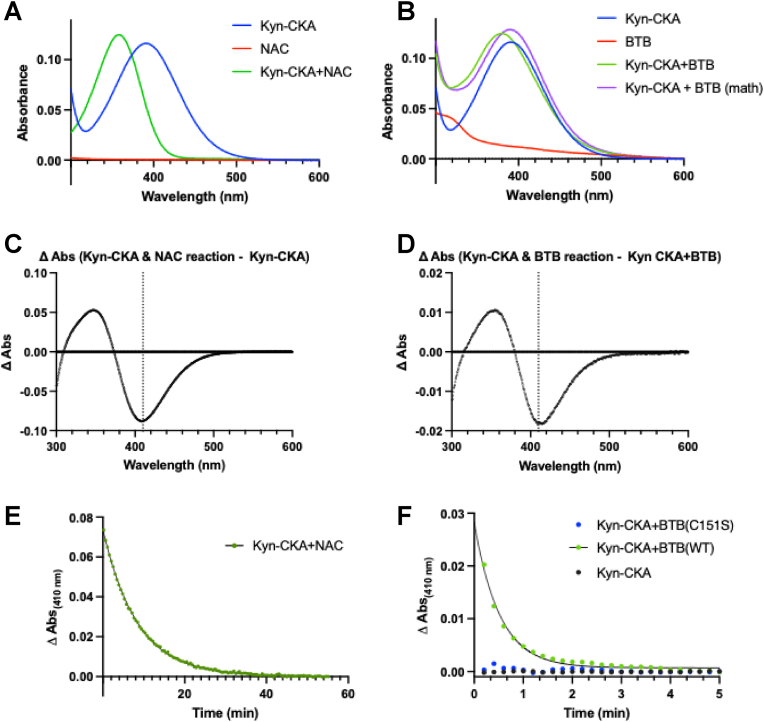
**The reaction of Kyn-CKA with Keap1 C151 is catalyzed by surrounding residues in the BTB domain. (A)** Absorbance spectra at pH 7.5 for Kyn-CKA (30 μM), NAC (3.5 mM, 24 μM thiolate), and the equilibrium product of their reaction. **(B)** Absorbance spectra at pH 7.5 for Kyn-CKA (30 μM), WT BTB (30 μM, 24 μM thiolate), and the equilibrium product of their reaction. The mathematical addition of the Kyn-CKA and the BTB spectra is shown in purple for comparison with the product of their reaction, in green. **(C)** The absorbance of the Kyn-CKA and NAC reaction at equilibrium (product, green trace in (A)) minus the absorbance of Kyn-CKA alone (reactant, blue trace in (A)) is shown. The vertical dotted line marks 410 nm on the x-axis. **(D)** The absorbance of the Kyn-CKA and BTB reaction at equilibrium (product, green trace in (B)) minus the absorbance of Kyn-CKA alone and BTB alone (reactants, purple trace in (B)) is shown. The vertical dotted line marks 410 nm on the x-axis. **(E and F)** The change in absorbance at 410 nm over time for each reaction was normalized to its absorbance at equilibrium, see method section for further details. Data for the reactions with NAC and WT BTB were fit to a one-phase decay equation.

Finally, α,β-unsaturated carbonyl Nrf2 activators have recently been shown to be targeted to Keap1 C151 by a hydrophobic active site pocket, which (i) orients the electrophilic carbon within reaction distance of the C151 thiolate for catalysis-by-proximity and (ii) establishes hydrogen bonding to the oxygen of the α,β-unsaturated carbonyl, which could both increase carbon electrophilicity and stabilize the negative charge in the transition state [9]. Spectroscopic examination of the reaction of Kyn-CKA with the small molecule thiol NAC at a slight molar excess of Kyn-CKA to thiolate (deprotonated NAC at this pH) caused a blue shift in peak absorbance, from 391 to 355 nm (Fig. 6A). A parallel experiment with purified recombinant protein containing the BTB domain of Keap1, in which C151 resides, showed similar spectral changes upon incubation with a slight molar excess of Kyn-CKA to thiolate C151, with peak absorbance of Kyn-CKA alone shifting to 378 nm for the conjugate (Fig. 6B). As the Keap1-BTB protein, unlike NAC, has absorbance in this region, the Kyn-CKA and Keap1-BTB spectra were mathematically added to illustrate the “pre-reaction” spectrum, with a maximum of 386 nm. Because of the relatively small blue shift in peak maxima for this reaction (∼5 nm), difference spectra were obtained (Fig. 6C and D). The largest difference between reactants and products was near 410 nm for both NAC and Keap1-BTB. Monitoring kinetics by the change at 410 nm, the reaction of Kyn-CKA with NAC came to equilibrium in approximately 40 min (Fig. 6E), whereas the reaction of Kyn-CKA with WT Keap1-BTB was complete in 2–3 min (Fig. 6F). Both fit to first-order kinetics. Crucially, the Keap1-BTB C151S mutant showed no reaction with Kyn-CKA (Fig. 6F). Given that the conjugation of Kyn-CKA to Keap1-BTB-C151 is much faster than its reaction with NAC, the residues surrounding C151 likely serve to catalyze the reaction of Kyn-CKA with C151, as has been previously reported for other α,β-unsaturated carbonyls [9,12].

**Fig. 7:**
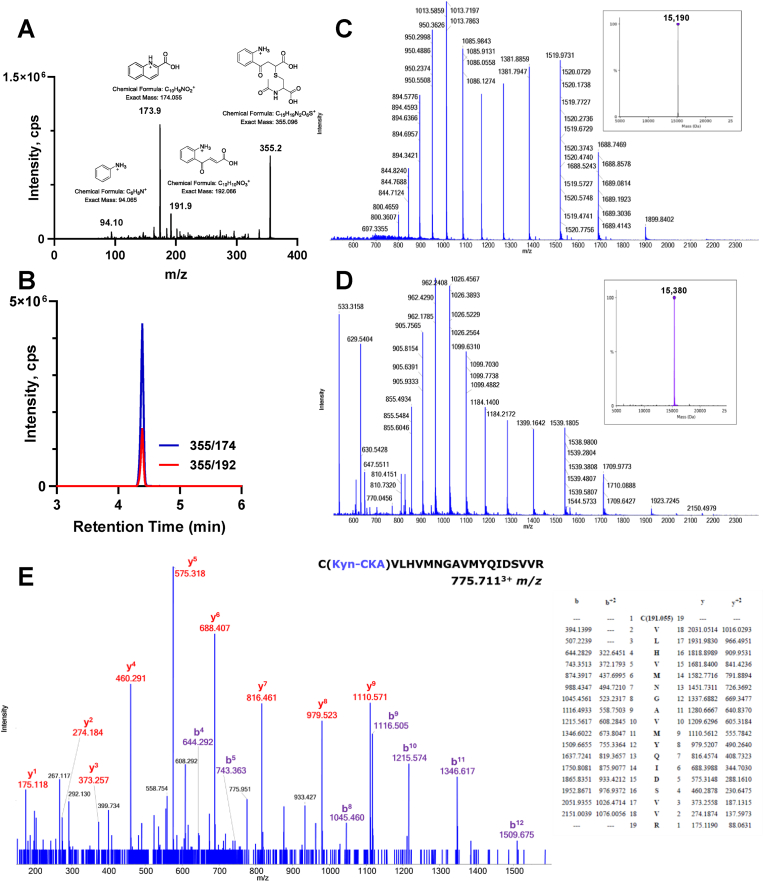
**LC-MS/MS characterization of the products of the reaction between Kyn-CKA, NAC and Keap1-BTB. (A**–**B)** Fragmentation spectrum and multiple reaction monitoring analysis of the final product of the reaction between Kyn-CKA and NAC under the same conditions as in 6A demonstrating the formation of the NAC-Kyn-CKA adduct. **(C**–**D)** High-resolution mass spectrum of the charge state distribution of the unreacted (C) and Kyn-CKA-reacted (D) Keap1-BTB segment and calculated *m*/*z* of the intact protein (inserts). **(E)** Collision-induced dissociation (CID) spectrum of C(Kyn-CKA)VLHVMNGAVMYQIDSVVR demonstrating the addition of the Kyn-CKA to Keap1-BTB-C151 with the series of b-ions showing the shift of 191.058 *m*/*z*.

The formation of the addition product NAC-Kyn-CKA from the reaction between Kyn-CKA and NAC was confirmed by LC-MS/MS analysis (Fig. 7A and B). In addition, high resolution MS/MS analysis of the product of the reaction between Kyn-CKA and Keap1-BTB demonstrated the modification of a single cysteine residue in the Keap1-BTB protein (Fig. 7C–D). No double-modified Keap1-BTB protein was detected (data not shown). While Keap1-BTB-C151 was found to be modified by Kyn-CKA as expected (Fig. 7E), we also found evidence of Kyn-CKA modification on C77 and C171 (Suppl. Fig. 1). Given that the loss of C151 completely inhibits the reaction between Kyn-CKA and Keap1 BTB (Fig. 6F), and that the BTB protein is modified at a single cysteine, the modifications of C77 and C171 are likely to represent additional minor products. Collectively, our results establish C151 in Keap1 as the principal sensor mediating Nrf2 activation and induction of its target genes in response to Kyn-CKA.

### 3.5 Kyn-CKA activates AhR, but this ligand-activated transcription factor is dispensable for the anti-inflammatory effects of Kyn-CKA

**Fig. 8:**
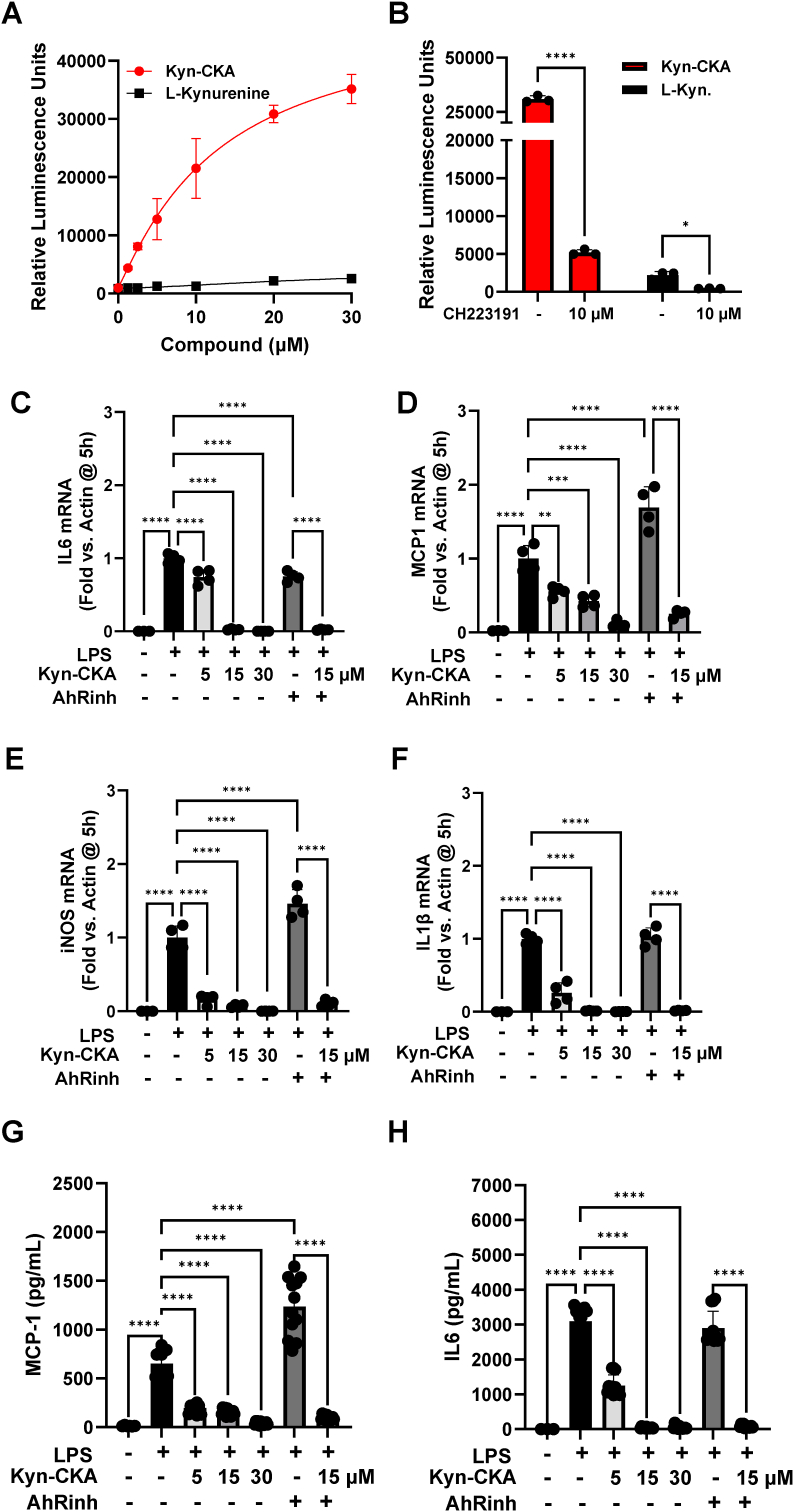
**AhR inhibition has no effect on the anti-inflammatory actions of Kyn-CKA in BMDMs. (A)** Mouse AhR reporter cells expressing luciferase under the control of the xenobiotic response element (XRE) were incubated with Kyn-CKA or kynurenine for 24 h prior to luminescence measurement (n = 3). Data were fitted to a sigmoidal four-parameter logistic curve (L-Kyn: r^2^ = 0.824, IC_50_ = 28 μM, span = 3218 RFU. Kyn-CKA: r^2^ = 0.970, IC_50_ = 13 μM, span = 47,901 RFU). **(B)** Inhibition of AhR-dependent luciferase expression by CH223191 (AhRinh). **(C–F)** Kyn-CKA inhibits the expression of the NF-κB-regulated genes IL6, MCP1, Nos2 and IL1β in BMDM following 5 h incubation with LPS (0.5 μg/mL) and the indicated concentrations of Kyn-CKA both in the presence or absence of CH223191 (10 μM). Data are n = 3–4, ∗∗∗∗p < 0.0001 by one-way ANOVA and Tukey's post-test. **(G**–**H)** Extracellular cytokine levels from BMDM treated as in C–F for 5 h. Data are n = 3–4 (all replicates shown), ∗∗∗∗p < 0.0001 by one-way ANOVA and Tukey's post-test.

**Fig. 9:**
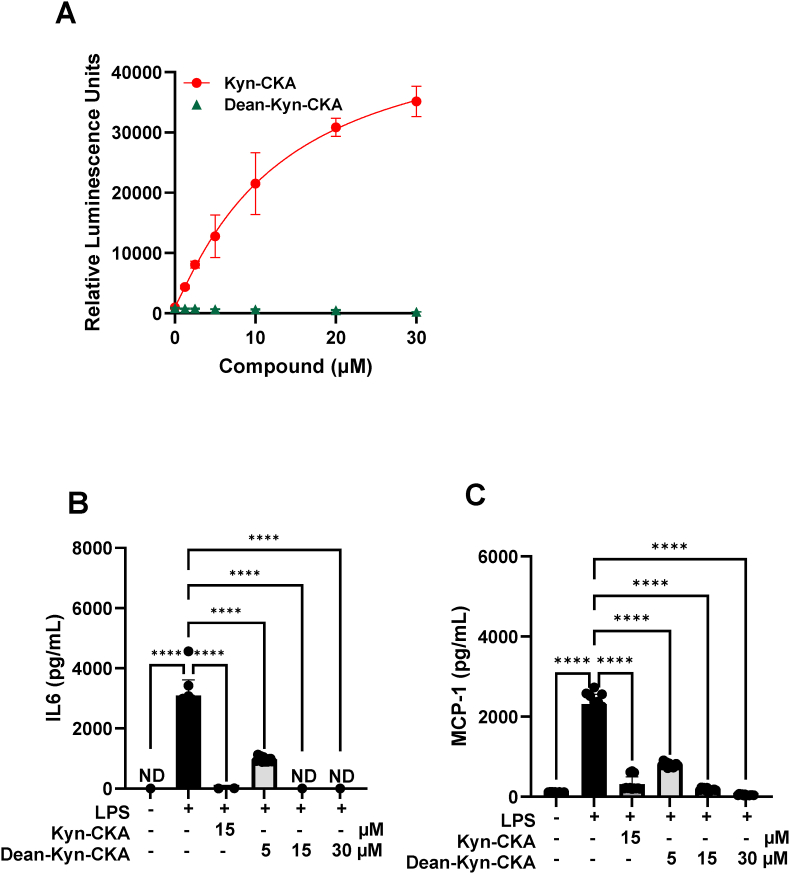
**The AhR-inactive metabolite Dean-CKA inhibits LPS-induced pro-inflammatory gene expression with similar potency to Kyn-CKA. (A)** Mouse AhR reporter cells expressing luciferase under control of the xenobiotic response element (XRE) were incubated with Dean-Kyn-CKA or Kyn-CKA for 24 h prior to luminescence measurement (n = 3). Data were fitted as in Fig. 8 (Dean-Kyn-CKA: No fit. Kyn-CKA: r^2^ = 0.970, IC_50_ = 13 μM, span = 47,901 RFU). **(B–C)** Extracellular cytokine levels from BMDM treated as in C–F for 5 h. Data are n = 3–4, ∗∗∗∗p < 0.0001 by one-way ANOVA and Tukey's post-test.

In addition to Nrf2, Kyn-CKA induces AhR-dependent gene expression while inhibiting NF-κB and NLRP3-dependent pro-inflammatory signalling [21]. The use of a mouse AhR reporter assay confirmed AhR activation by Kyn-CKA and further showed that Kyn-CKA is a more potent AhR activator than kynurenine following a 24 h incubation (Fig. 8A). In macrophages, AhR activation suppresses NF-κB transcriptional activity resulting in decreased TNFα and IL6 production [43,44]. Consistent with these observations, the chemical inhibition of AhR with CH223191 diminished AhR activity in the reporter assay (Fig. 8B) and resulted in a slight elevation in the mRNA levels for MCP1 and Nos2 upon BMDM challenge with LPS. However, CH223191 had no effect on the ability of Kyn-CKA to suppress the LPS-mediated pro-inflammatory responses in these cells (Fig. 8C–F). In agreement with the mRNA data, AhR inhibition did not affect the ability of Kyn-CKA to decrease the secreted levels of MCP1 and IL6 in the cell culture media (Fig. 8G and H). Moreover, the deaminated derivative of Kyn-CKA, Dean-CKA, which activated Nrf2 (Fig. 3A), but did not activate AhR (Fig. 9A), also lowered the levels of secreted IL6 and MCP1 from LPS-stimulated BMDMs (Fig. 9B and C), further indicating that activation of AhR is not required for the acute anti-inflammatory activity of compounds of this type.

**Fig. 10:**
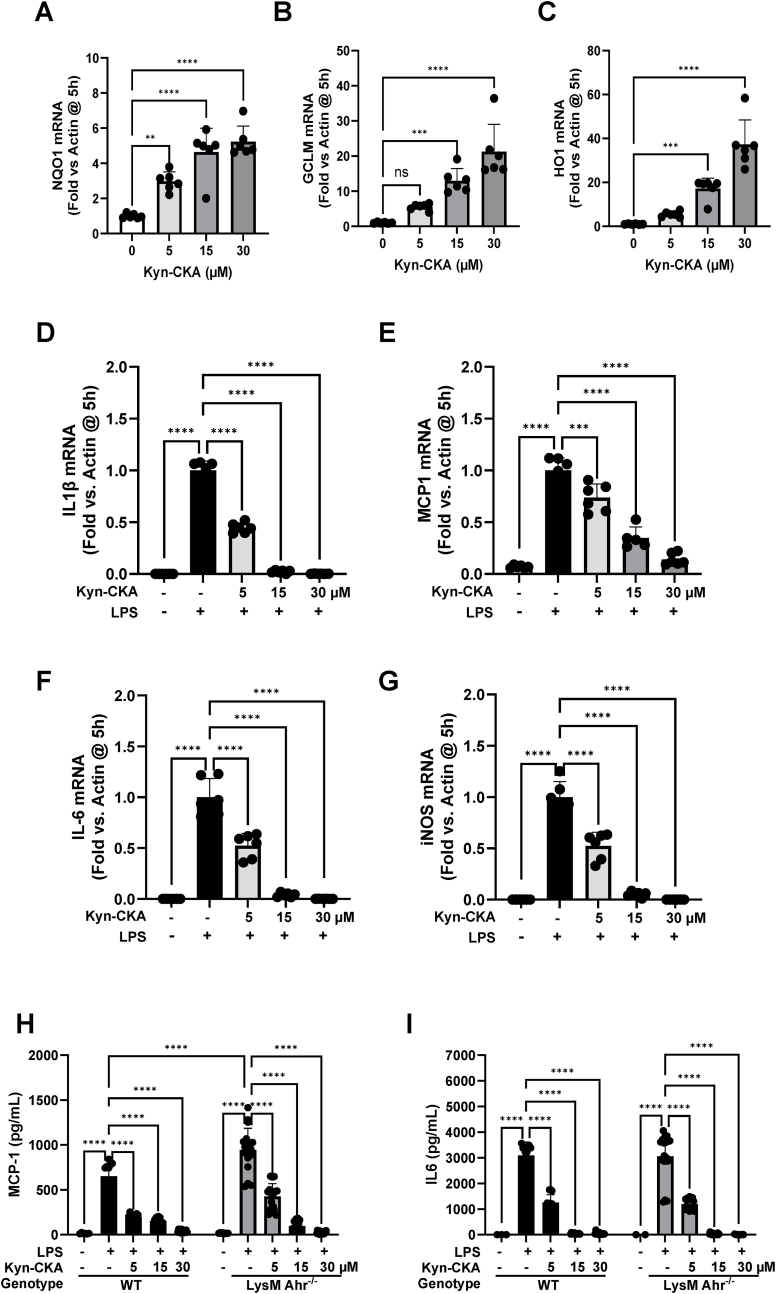
**Kyn-CKA inhibits LPS-elicited responses in AhR-knockout BMDMs. (A**–**C)** Kyn-CKA dose-dependently induces Nrf2-dependent gene expression in BMDM obtained from LysM AhR-knockout (AhR^−/−^) mice. Data are n = 6, ∗∗p < 0.01, ∗∗∗p < 0.001, ∗∗∗∗p < 0.0001 by one-way ANOVA and Dunnett's post-test. **(D**–**G)** Kyn-CKA inhibits the expression of NF-κB-regulated genes in LysM AhR^−/−^ BMDM following 5h incubation with LPS (0.5 μg/mL) and the indicated concentrations of Kyn-CKA. Data are n = 6, ∗∗∗∗p < 0.0001 by one-way ANOVA and Dunnett's post-test. **(H–I)** Extracellular cytokine levels from WT and LysM AhR^−/−^ BMDM treated as in D-G for 5 h. Data are n = 6, ∗∗∗∗p < 0.0001 by two-way ANOVA and Tukey's post-test. The WT BMDM data were replotted from Fig. 8 G–H for comparison purposes.

Experiments in BMDMs derived from monocyte-specific AhR-knockout (*LysM Ahr*^*−/−*^) mice confirmed that Kyn-CKA treatment leads to transcriptional upregulation of Nrf2-target genes (Fig. 10A–C), indicating that the absence of the AhR does not affect the ability of Kyn-CKA to activate Nrf2. Most importantly, these experiments further showed that the downregulation of the LPS-mediated transcription of pro-inflammatory genes by Kyn-CKA does not require the presence of the AhR (Fig. 10D–I). Together, these data establish firmly that the AhR is dispensable for the anti-inflammatory activity of Kyn-CKA in macrophages.

### 3.6 The ability of Kyn-CKA to suppress pro-inflammatory responses is partly Nrf2-dependent

**Fig. 11:**
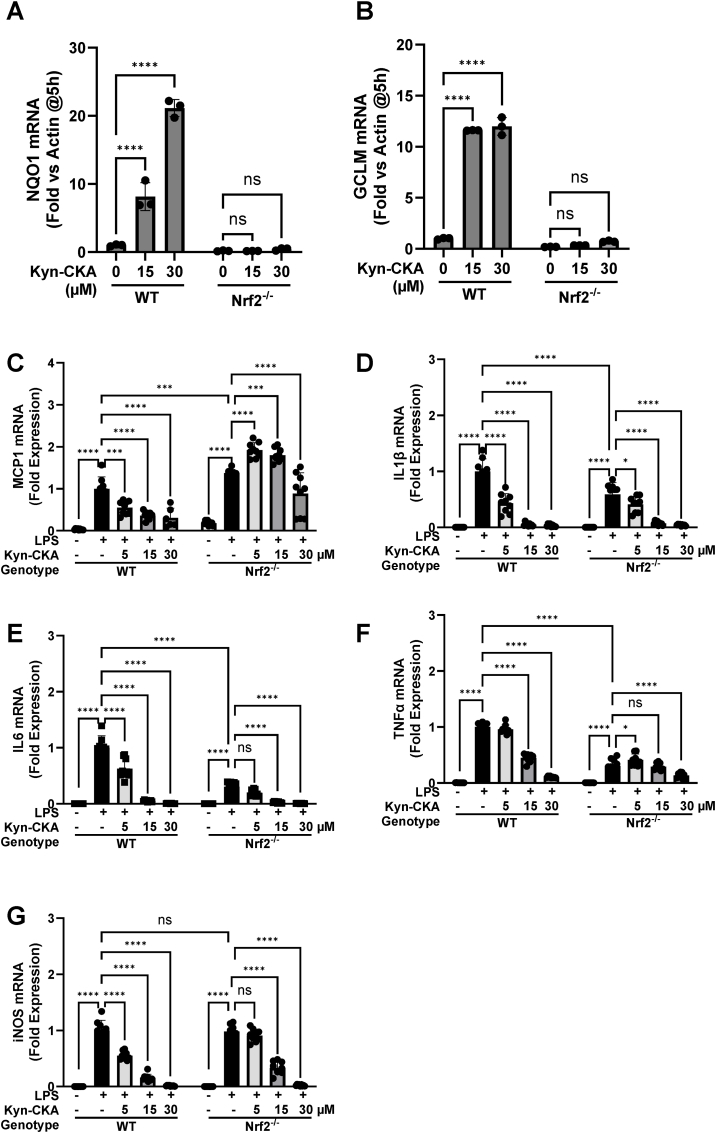
**The low-dose anti-inflammatory effects of Kyn-CKA in BMDMs are dependent on Nrf2. (A**–**B)** Treatment with Kyn-CKA (5 h) fails to induce the expression of *Nqo1* and *Gclm* in BMDMs obtained from Nrf2-knockout (Nrf2^−/−^) mice. Data are n = 3, ∗∗∗∗p < 0.0001 by two-way ANOVA and Tukey's post-test. **(C**–**G)** Effects of Kyn-CKA on the expression of NF-κB−regulated genes in WT and Nrf2-KO BMDMs following 5 h incubation with LPS (0.5 μg/mL) and the indicated concentrations of Kyn-CKA. Data are n = 6, ∗p < 0.05, ∗∗∗p < 0.001, ∗∗∗∗p < 0.0001 by two-way ANOVA and Tukey's post-test. Nrf2 was identified as a significant source of variation for MCP1, IL6, TNFα, Nos2, (p < 0.0001) and IL1β (p < 0.005).

To test whether Nrf2 contributes to the anti-inflammatory activity of Kyn-CKA, we used BMDMs from WT and Nrf2-KO mice. Nrf2 activation by Kyn-CKA was confirmed by the increase in mRNA levels for Nqo1 and Gclm, which occurred in WT, but not Nrf2-KO cells (Fig. 11A and B). As expected, Kyn-CKA treatment of WT BMDMs, led to a concentration-dependent downregulation of the LPS-stimulated transcription of the pro-inflammatory markers MCP1, IL1β, IL6, TNFα and Nos2 (Fig. 11C–G). In contrast, the suppressive effect of the low (5 μM) concentration of Kyn-CKA was not apparent in Nrf2-KO BMDMs but was still observed when higher Kyn-CKA concentrations were used; this effect was particularly evident at the highest (30 μM) concentration of Kyn-CKA. Together, these experiments suggest that, at low concentrations, Keap1/Nrf2 mediate the anti-inflammatory effect of Kyn-CKA, but at high concentrations, Kyn-CKA also affects other proteins that are involved in the inflammatory response.

## 4 Discussion

Keap1 was discovered ∼25 years ago as the principal negative regulator of Nrf2 [45]. Shortly afterwards, it was demonstrated that Keap1 is endowed with reactive cysteines that function as molecular sensors for a wide variety of small molecules, most of them xenobiotics, all of which have sulfhydryl reactivity as their common chemical property [46]. More recently, several endogenous electrophiles have been shown to modify cysteines in Keap1; these have been reviewed [16]. Such modifications inactivate the substrate adaptor activity of Keap1, resulting in de-repression of Nrf2 and induction of a broad cytoprotective program that allows adaptation and survival under conditions of cellular stress, restoring homeostasis. One putative endogenous Nrf2 activator is the tryptophan metabolite kynurenine [33]. However, kynurenine lacks electrophilic reactivity, and the mechanism by which it activates Nrf2 was not known hitherto. Here, we confirm that kynurenine is a low-potency (high micromolar range) activator of Nrf2. We further show that its electrophilic metabolite Kyn-CKA is more potent than its kynurenine precursor and induces Nrf2-mediated transcription, most likely through reacting with C151 within Keap1. Together with our previous report that kynurenine synthesis in cells, such as macrophages, leads to Kyn-CKA formation and that Kyn-CKA–specific metabolites have been detected in humans and mice [21], the findings from the current study strongly suggest that Kyn-CKA rather than kynurenine, is the *de facto* inducer.

Our biochemical experiments with recombinant Keap1-BTB protein and its C151S mutant counterpart (Fig. 6) suggest that C151 in Keap1 is a sensor for Kyn-CKA. This notion is further supported by the high resolution MS/MS analyses of the reaction product of Kyn-CKA and Keap1-BTB showing the modification of a single cysteine in Keap1-BTB (Fig. 7), and by experiments in C151S Keap1 mutant cells, where the ability of Kyn-CKA to upregulate Nrf2 or NQO1 is impaired, in stark contrast with the upregulation of both Nrf2 and NQO1 in cells expressing wild-type or any of the other cysteine sensor mutants of Keap1 tested (Fig. 5). Nonetheless, it is possible that, at higher Kyn-CKA concentrations, or at specific reaction conditions, multiple cysteines in Keap1 are modified. Notably, such additional cysteine modifications have been reported for other electrophilic C151-targeting Keap1 inhibitors, such as TBE-31 [10] and SFN [47], and highlights the intrinsic flexibility of Keap1, where cysteine sensors can be engaged redundantly in a “fail-safe” mechanism to allow Nrf2 activation, preventing electrophile-mediated glutathione depletion and oxidative stress to ensure adaptation and survival. Alternatively, it is possible that in cells, Keap1 could be modified by an unknown electrophilic metabolite of Kyn-CKA and/or subsequent generation of reactive oxygen species via redox cycling and glutathione depletion; however, this latter possibility is less likely as H_2_O_2_ sensing occurs through combinations of C226, C613, *C*222 and/or C624 in Keap1 [27].

In macrophages, genetic or pharmacological Nrf2 activation, including by Kyn-CKA, suppresses pro-inflammatory responses [21]. In addition to Nrf2, kynurenine and Kyn-CKA promote AhR-dependent gene expression [21]. Activation of AhR has anti-inflammatory effects; and this has been shown in animal models of human disease, such as autoimmune encephalomyelitis and retinal ischemia/reperfusion injury [48,49]. Thus, in this study we considered the possibility that the anti-inflammatory effects of Kyn-CKA might be mediated, at least in part, by AhR. The use of BMDMs derived from monocyte-specific AhR-knockout mice revealed that, although Kyn-CKA robustly activates AhR, the anti-inflammatory effect of this metabolite is AhR-independent. In contrast, our experiments in BMDMs derived from Nrf2-KO mice showed a clear Nrf2-dependence at low Kyn-CKA concentrations that likely reflect its endogenous cellular levels [21].

Interestingly, in LPS-stimulated BMDM cells, the expression of IL1β, IL6 and TNFα was lower in the absence of Nrf2. This finding is in close agreement with our earlier results in SKH1- hairless mice exposed to solar-simulated ultraviolet radiation (SSUV) showing that, whereas Nrf2 is required for the anti-inflammatory effect of TBE-31, the SSUV-induced expression of IL1β and IL6 in the skin of Nrf2-KO mice was lower than in the skin of their wild-type counterparts [24]. A recent RNA-seq analysis has revealed that Nrf2 deficiency in microglia dampens the microglial pro-inflammatory responses to systemic LPS administration [50]. Further investigations are necessary to elucidate the underlying mechanism(s), but these observations strongly suggest that, in addition to its widely recognized role in the resolution of inflammation, Nrf2 might also support the inflammatory response.

Notably, at high concentrations, Kyn-CKA was still able to suppress the transcription of pro-inflammatory genes in the absence of Nrf2. These results are consistent with our previous findings in murine peritoneal macrophages stimulated with IFNγ and TNFα, where two potent Nrf2 activators (SFN and the pentacyclic cyanoenone TP-225) were much less effective inhibitors of Nos2 induction in Nrf2-KO in comparison with WT cells, but inhibition still occurred when higher concentrations of these electrophiles were used [15]. We suggest that the Nrf2-independent effects of Kyn-CKA are mediated by inhibition of NF-κB signalling potentially associated with direct covalent modification of p65 [21], but that the Keap1/Nrf2 system, which is affected by the low concentrations of Kyn-CKA, is the primary target. This scenario is reminiscent of the ‘‘hierarchical’’ or ‘‘rheostat’’ response of the murine macrophage cell line RAW 264.7 to diesel exhaust particles, which contain redox cycling organic chemicals that induce pro-oxidative and pro-inflammatory effects [51]. In that response, the Keap1/Nrf2 system is the first-tier defence (activated by modest increases of reactive oxygen/nitrogen species (ROS/RNS), whereas NF-κB represents the second-tier defence (activated by higher ROS/RNS levels); the third and final tier involves activation of apoptosis. Thus, as described for conditions of oxidative stress [51], the Keap1/Nrf2 system may serve as a ‘‘floodgate’’ for electrophiles, such as Kyn-CKA, in which other transcription factors are affected at a particular threshold of concentration only after “saturation” of the Keap1 cysteine sensor(s).

## CRediT authorship contribution statement

**Jialin Feng:** Data curation, Formal analysis, Investigation, Writing – review & editing. **Mara Carreño:** Data curation, Formal analysis, Investigation. **Hannah Jung:** Data curation, Formal analysis, Investigation. **Sharadha Dayalan Naidu:** Methodology, Supervision. **Nicole Arroyo-Diaz:** Investigation, Methodology. **Abel D. Ang:** Resources. **Bhargavi Kulkarni:** Investigation. **Dorothy Kisielewski:** Resources. **Takafumi Suzuki:** Resources, Writing – review & editing. **Masayuki Yamamoto:** Writing – review & editing. **John D. Hayes:** Writing – review & editing. **Tadashi Honda:** Resources. **Landon Wilson:** Formal analysis, Investigation. **Beatriz Leon-Ruiz:** Resources. **Aimee L. Eggler:** Conceptualization, Funding acquisition, Supervision, Writing – review & editing. **Dario A. Vitturi:** Conceptualization, Data curation, Formal analysis, Funding acquisition, Writing – review & editing. **Albena T. Dinkova-Kostova:** Conceptualization, Data curation, Formal analysis, Funding acquisition, Supervision, Writing – original draft, Writing – review & editing.

## Declaration of competing interest

The authors declare that they have no known competing financial interests or personal relationships that could have appeared to influence the work reported in this paper.

## Data Availability

Data will be made available on request.

## References

[1] Yamamoto M., Kensler T.W., Motohashi H. (2018). The KEAP1-NRF2 system: a thiol-based sensor-effector apparatus for maintaining redox homeostasis. Physiol. Rev..

[2] Hayes J.D., Dayalan Naidu S., Dinkova-Kostova A.T. (2025). Regulating Nrf2 activity: ubiquitin ligases and signaling molecules in redox homeostasis. Trends Biochem. Sci..

[3] Thimmulappa R.K., Lee H., Rangasamy T., Reddy S.P., Yamamoto M., Kensler T.W., Biswal S. (2006). Nrf2 is a critical regulator of the innate immune response and survival during experimental sepsis. J. Clin. Investig..

[4] Mills E.L., Ryan D.G., Prag H.A., Dikovskaya D., Menon D., Zaslona Z., Jedrychowski M.P., Costa A.S.H., Higgins M., Hams E., Szpyt J., Runtsch M.C., King M.S., McGouran J.F., Fischer R., Kessler B.M., McGettrick A.F., Hughes M.M., Carroll R.G., Booty L.M., Knatko E.V., Meakin P.J., Ashford M.L.J., Modis L.K., Brunori G., Sevin D.C., Fallon P.G., Caldwell S.T., Kunji E.R.S., Chouchani E.T., Frezza C., Dinkova-Kostova A.T., Hartley R.C., Murphy M.P., O'Neill L.A. (2018). Itaconate is an anti-inflammatory metabolite that activates Nrf2 via alkylation of KEAP1. Nature.

[5] Kobayashi E.H., Suzuki T., Funayama R., Nagashima T., Hayashi M., Sekine H., Tanaka N., Moriguchi T., Motohashi H., Nakayama K., Yamamoto M. (2016). Nrf2 suppresses macrophage inflammatory response by blocking proinflammatory cytokine transcription. Nat. Commun..

[6] Cuadrado A., Rojo A.I., Wells G., Hayes J.D., Cousin S.P., Rumsey W.L., Attucks O.C., Franklin S., Levonen A.L., Kensler T.W., Dinkova-Kostova A.T. (2019). Therapeutic targeting of the NRF2 and KEAP1 partnership in chronic diseases. Nat. Rev. Drug Discov..

[7] Suzuki T., Yamamoto M. (2015). Molecular basis of the Keap1-Nrf2 system. Free Radic. Biol. Med..

[8] McMahon M., Lamont D.J., Beattie K.A., Hayes J.D. (2010). Keap1 perceives stress via three sensors for the endogenous signaling molecules nitric oxide, zinc, and alkenals. Proc. Natl. Acad. Sci. U. S. A..

[9] Schnell M.R., Zhai T., Ragwan E.R., Jung H., Zhang J., Lagalante A.F., Kung Y., Kraut D.A., Huang Z., Eggler A.L. (2025). KEAP1 C151 active site catalysis drives electrophilic signaling to upregulate cytoprotective enzyme expression. Redox Biol..

[10] Dayalan Naidu S., Muramatsu A., Saito R., Asami S., Honda T., Hosoya T., Itoh K., Yamamoto M., Suzuki T., Dinkova-Kostova A.T. (2018). C151 in KEAP1 is the main cysteine sensor for the cyanoenone class of NRF2 activators, irrespective of molecular size or shape. Sci. Rep..

[11] Zhang D.D., Hannink M. (2003). Distinct cysteine residues in Keap1 are required for Keap1-dependent ubiquitination of Nrf2 and for stabilization of Nrf2 by chemopreventive agents and oxidative stress. Mol. Cell Biol..

[12] Sauerland M., Mertes R., Morozzi C., Eggler A.L., Gamon L.F., Davies M.J. (2021). Kinetic assessment of Michael addition reactions of alpha, beta-unsaturated carbonyl compounds to amino acid and protein thiols. Free Radic. Biol. Med..

[13] Shekh-Ahmad T., Eckel R., Dayalan Naidu S., Higgins M., Yamamoto M., Dinkova-Kostova A.T., Kovac S., Abramov A.Y., Walker M.C. (2018). KEAP1 inhibition is neuroprotective and suppresses the development of epilepsy. Brain.

[14] Dinkova-Kostova A.T., Liby K.T., Stephenson K.K., Holtzclaw W.D., Gao X., Suh N., Williams C., Risingsong R., Honda T., Gribble G.W., Sporn M.B., Talalay P. (2005). Extremely potent triterpenoid inducers of the phase 2 response: correlations of protection against oxidant and inflammatory stress. Proc. Natl. Acad. Sci. U. S. A..

[15] Liu H., Dinkova-Kostova A.T., Talalay P. (2008). Coordinate regulation of enzyme markers for inflammation and for protection against oxidants and electrophiles. Proc. Natl. Acad. Sci. U. S. A..

[16] Dinkova-Kostova A.T., Hakomaki H., Levonen A.L. (2024). Electrophilic metabolites targeting the KEAP1/NRF2 partnership. Curr. Opin. Chem. Biol..

[17] Takenaka M.C., Gabriely G., Rothhammer V., Mascanfroni I.D., Wheeler M.A., Chao C.C., Gutierrez-Vazquez C., Kenison J., Tjon E.C., Barroso A., Vandeventer T., de Lima K.A., Rothweiler S., Mayo L., Ghannam S., Zandee S., Healy L., Sherr D., Farez M.F., Prat A., Antel J., Reardon D.A., Zhang H., Robson S.C., Getz G., Weiner H.L., Quintana F.J. (2019). Control of tumor-associated macrophages and T cells in glioblastoma via AHR and CD39. Nat. Neurosci..

[18] Opitz C.A., Holfelder P., Prentzell M.T., Trump S. (2023). The complex biology of aryl hydrocarbon receptor activation in cancer and beyond. Biochem. Pharmacol..

[19] Miao W., Hu L., Scrivens P.J., Batist G. (2005). Transcriptional regulation of NF-E2 p45-related factor (NRF2) expression by the aryl hydrocarbon receptor-xenobiotic response element signaling pathway: direct cross-talk between phase I and II drug-metabolizing enzymes. J. Biol. Chem..

[20] Huchzermeier R., van der Vorst E.P.C. (2025). Aryl hydrocarbon receptor (AHR) and nuclear factor erythroid-derived 2-like 2 (NRF2): an important crosstalk in the gut-liver axis. Biochem. Pharmacol..

[21] Carreno M., Pires M.F., Woodcock S.R., Brzoska T., Ghosh S., Salvatore S.R., Chang F., Khoo N.K.H., Dunn M., Connors N., Yuan S., Straub A.C., Wendell S.G., Kato G.J., Freeman B.A., Ofori-Acquah S.F., Sundd P., Schopfer F.J., Vitturi D.A. (2022). Immunomodulatory actions of a kynurenine-derived endogenous electrophile. Sci. Adv..

[22] Fahrmann J.F., Tanaka I., Irajizad E., Mao X., Dennison J.B., Murage E., Casabar J., Mayo J., Peng Q., Celiktas M., Vykoukal J.V., Park S., Taguchi A., Delgado O., Tripathi S.C., Katayama H., Soto L.M.S., Rodriguez-Canales J., Behrens C., Wistuba I., Hanash S., Ostrin E.J. (2022). Mutational activation of the NRF2 pathway upregulates kynureninase resulting in tumor immunosuppression and poor outcome in lung adenocarcinoma. Cancers (Basel).

[23] Saito A Z.S., Takahashi M., Li W., Ojima I., Honda T. (2013). An improved synthesis of a hydroxymethyl tricyclic ketone from cyclohexanone, the key process for the synthesis of a highly potent anti-inflammatory and cytoprotective agent. Synthesis.

[24] Knatko E.V., Ibbotson S.H., Zhang Y., Higgins M., Fahey J.W., Talalay P., Dawe R.S., Ferguson J., Huang J.T., Clarke R., Zheng S., Saito A., Kalra S., Benedict A.L., Honda T., Proby C.M., Dinkova-Kostova A.T. (2015). Nrf2 activation protects against solar-simulated ultraviolet radiation in mice and humans. Cancer Prev. Res..

[25] Itoh K., Chiba T., Takahashi S., Ishii T., Igarashi K., Katoh Y., Oyake T., Hayashi N., Satoh K., Hatayama I., Yamamoto M., Nabeshima Y. (1997). An Nrf2/small Maf heterodimer mediates the induction of phase II detoxifying enzyme genes through antioxidant response elements. Biochem. Biophys. Res. Commun..

[26] Taguchi K., Maher J.M., Suzuki T., Kawatani Y., Motohashi H., Yamamoto M. (2010). Genetic analysis of cytoprotective functions supported by graded expression of Keap1. Mol. Cell Biol..

[27] Suzuki T., Muramatsu A., Saito R., Iso T., Shibata T., Kuwata K., Kawaguchi S.I., Iwawaki T., Adachi S., Suda H., Morita M., Uchida K., Baird L., Yamamoto M. (2019). Molecular mechanism of cellular oxidative stress sensing by Keap1. Cell Rep..

[28] Dayalan Naidu S., Suzuki T., Dikovskaya D., Knatko E.V., Higgins M., Sato M., Novak M., Villegas J.A., Moore T.W., Yamamoto M., Dinkova-Kostova A.T. (2022). The isoquinoline PRL-295 increases the thermostability of Keap1 and disrupts its interaction with Nrf2. iScience.

[29] Li B., Cui W., Liu J., Li R., Liu Q., Xie X.H., Ge X.L., Zhang J., Song X.J., Wang Y., Guo L. (2013). Sulforaphane ameliorates the development of experimental autoimmune encephalomyelitis by antagonizing oxidative stress and Th17-related inflammation in mice. Exp. Neurol..

[30] Dayalan Naidu S., Dikovskaya D., Moore T.W., Dinkova-Kostova A.T. (2022). Detection of thermal shift in cellular Keap1 by protein-protein interaction inhibitors using immunoblot- and fluorescence microplate-based assays. STAR Protoc..

[31] Ryan D.G., Knatko E.V., Casey A.M., Hukelmann J.L., Dayalan Naidu S., Brenes A.J., Ekkunagul T., Baker C., Higgins M., Tronci L., Nikitopolou E., Honda T., Hartley R.C., O'Neill L.A.J., Frezza C., Lamond A.I., Abramov A.Y., Arthur J.S.C., Cantrell D.A., Murphy M.P., Dinkova-Kostova A.T. (2022). Nrf2 activation reprograms macrophage intermediary metabolism and suppresses the type I interferon response. iScience.

[32] Fahey J.W., Dinkova-Kostova A.T., Stephenson K.K., Talalay P. (2004). The "Prochaska" microtiter plate bioassay for inducers of NQO1. Methods Enzymol..

[33] Fiore A., Zeitler L., Russier M., Gross A., Hiller M.K., Parker J.L., Stier L., Kocher T., Newstead S., Murray P.J. (2022). Kynurenine importation by SLC7A11 propagates anti-ferroptotic signaling. Mol. Cell.

[34] Talalay P., De Long M.J., Prochaska H.J. (1988). Identification of a common chemical signal regulating the induction of enzymes that protect against chemical carcinogenesis. Proc. Natl. Acad. Sci. U. S. A..

[35] Dinkova-Kostova A.T., Massiah M.A., Bozak R.E., Hicks R.J., Talalay P. (2001). Potency of Michael reaction acceptors as inducers of enzymes that protect against carcinogenesis depends on their reactivity with sulfhydryl groups. Proc. Natl. Acad. Sci. U. S. A..

[36] Gao X., Dinkova-Kostova A.T., Talalay P. (2001). Powerful and prolonged protection of human retinal pigment epithelial cells, keratinocytes, and mouse leukemia cells against oxidative damage: the indirect antioxidant effects of sulforaphane. Proc. Natl. Acad. Sci. U. S. A..

[37] Deighton R.F., Markus N.M., Al-Mubarak B., Bell K.F., Papadia S., Meakin P.J., Chowdhry S., Hayes J.D., Hardingham G.E. (2014). Nrf2 target genes can be controlled by neuronal activity in the absence of Nrf2 and astrocytes. Proc. Natl. Acad. Sci. U. S. A..

[38] Dinkova-Kostova A.T., Kostov R.V., Canning P. (2016). Keap1, the cysteine-based mammalian intracellular sensor for electrophiles and oxidants. Arch. Biochem. Biophys..

[39] Jafari R., Almqvist H., Axelsson H., Ignatushchenko M., Lundback T., Nordlund P., Molina D.M. (2014). The cellular thermal shift assay for evaluating drug target interactions in cells. Nat. Protoc..

[40] Martinez Molina D., Jafari R., Ignatushchenko M., Seki T., Larsson E.A., Dan C., Sreekumar L., Cao Y., Nordlund P. (2013). Monitoring drug target engagement in cells and tissues using the cellular thermal shift assay. Science.

[41] Cvetko F., Caldwell S.T., Higgins M., Suzuki T., Yamamoto M., Prag H.A., Hartley R.C., Dinkova-Kostova A.T., Murphy M.P. (2021). Nrf2 is activated by disruption of mitochondrial thiol homeostasis but not by enhanced mitochondrial superoxide production. J. Biol. Chem..

[42] Bollong M.J., Lee G., Coukos J.S., Yun H., Zambaldo C., Chang J.W., Chin E.N., Ahmad I., Chatterjee A.K., Lairson L.L., Schultz P.G., Moellering R.E. (2018). A metabolite-derived protein modification integrates glycolysis with KEAP1-NRF2 signalling. Nature.

[43] Kimura A., Naka T., Nakahama T., Chinen I., Masuda K., Nohara K., Fujii-Kuriyama Y., Kishimoto T. (2009). Aryl hydrocarbon receptor in combination with Stat1 regulates LPS-induced inflammatory responses. J. Exp. Med..

[44] Tian Y., Ke S., Denison M.S., Rabson A.B., Gallo M.A. (1999). Ah receptor and NF-kappaB interactions, a potential mechanism for dioxin toxicity. J. Biol. Chem..

[45] Itoh K., Wakabayashi N., Katoh Y., Ishii T., Igarashi K., Engel J.D., Yamamoto M. (1999). Keap1 represses nuclear activation of antioxidant responsive elements by Nrf2 through binding to the amino-terminal Neh2 domain. Genes Dev..

[46] Dinkova-Kostova A.T., Holtzclaw W.D., Cole R.N., Itoh K., Wakabayashi N., Katoh Y., Yamamoto M., Talalay P. (2002). Direct evidence that sulfhydryl groups of Keap1 are the sensors regulating induction of phase 2 enzymes that protect against carcinogens and oxidants. Proc. Natl. Acad. Sci. U. S. A..

[47] Hu C., Eggler A.L., Mesecar A.D., van Breemen R.B. (2011). Modification of keap1 cysteine residues by sulforaphane. Chem. Res. Toxicol..

[48] Rothhammer V., Mascanfroni I.D., Bunse L., Takenaka M.C., Kenison J.E., Mayo L., Chao C.C., Patel B., Yan R., Blain M., Alvarez J.I., Kebir H., Anandasabapathy N., Izquierdo G., Jung S., Obholzer N., Pochet N., Clish C.B., Prinz M., Prat A., Antel J., Quintana F.J. (2016). Type I interferons and microbial metabolites of tryptophan modulate astrocyte activity and central nervous system inflammation via the aryl hydrocarbon receptor. Nat. Med..

[49] Yang Y., Wang N., Xu L., Liu Y., Huang L., Gu M., Wu Y., Guo W., Sun H. (2023). Aryl hydrocarbon receptor dependent anti-inflammation and neuroprotective effects of tryptophan metabolites on retinal ischemia/reperfusion injury. Cell Death Dis..

[50] He X., Dando O., Qiu J. (2025). Nrf2 controls homeostatic transcriptional signatures and inflammatory responses in a cell-type specific manner in the adult mouse brain. iScience.

[51] Xiao G.G., Wang M., Li N., Loo J.A., Nel A.E. (2003). Use of proteomics to demonstrate a hierarchical oxidative stress response to diesel exhaust particle chemicals in a macrophage cell line. J. Biol. Chem..

