# Supplementary material for: The electrophilic metabolite of kynurenine, kynurenine-CKA, requires C151 in Keap1 to derepress Nrf2
Source: Redox Biol. 2026 Jan 6;90:104009. doi: 10.1016/j.redox.2026.104009 (PMC12828764; doi:10.1016/j.redox.2026.104009)
Supplement: Multimedia component 1 [file mmc1.pdf]

Supplemental Information

The electrophilic metabolite of kynurenine, kynurenine-CKA, requires C151 in Keap1 to derepress Nrf2

Jialin Feng[1*], Mara Carreño[2*], Hannah Jung[3], Sharadha Dayalan Naidu[1], Nicole Arroyo-Diaz[2], Abel D. Ang[1], Bhargavi Kulkarni[2], Dorothy Kisielewski[1], Takafumi Suzuki[4], Masayuki Yamamoto[4], John D. Hayes[1], Tadashi Honda[5], Landon Wilson[6], Beatriz Leon-Ruiz[7], Aimee L. Eggler[3], Dario A. Vitturi[2**], Albena T. Dinkova-Kostova[1,8**]

[1]Jacqui Wood Cancer Centre, Division of Cancer Research, School of Medicine, University of Dundee, Dundee, UK

[2]Department of Pathology, School of Medicine, The University of Alabama at Birmingham, Birmingham, AL, USA

[3]Department of Chemistry and Biochemistry, Villanova University, Villanova, PA, USA

[4]Department of Biochemistry and Molecular Biology, Tohoku Medical Megabank Organization, Tohoku University, Sendai, Japan

[5]Department of Chemistry and Institute of Chemical Biology & Drug Discovery, Stony Brook University, Stony Brook, NY, USA

[6]Targeted Metabolomics and Proteomics Laboratory, Department of Pharmacology and Toxicology, University of Alabama at Birmingham, AL, USA

[7]Innate Cells and Th2 Immunity Section, Laboratory of Allergic Diseases, National Institute of Allergy and Infectious Diseases, National Institutes of Health, Bethesda, MD, USA

[8]Department of Physiology, Pharmacology and Therapeutics and Department of Medicine, Johns Hopkins University School of Medicine, Baltimore, MD, USA

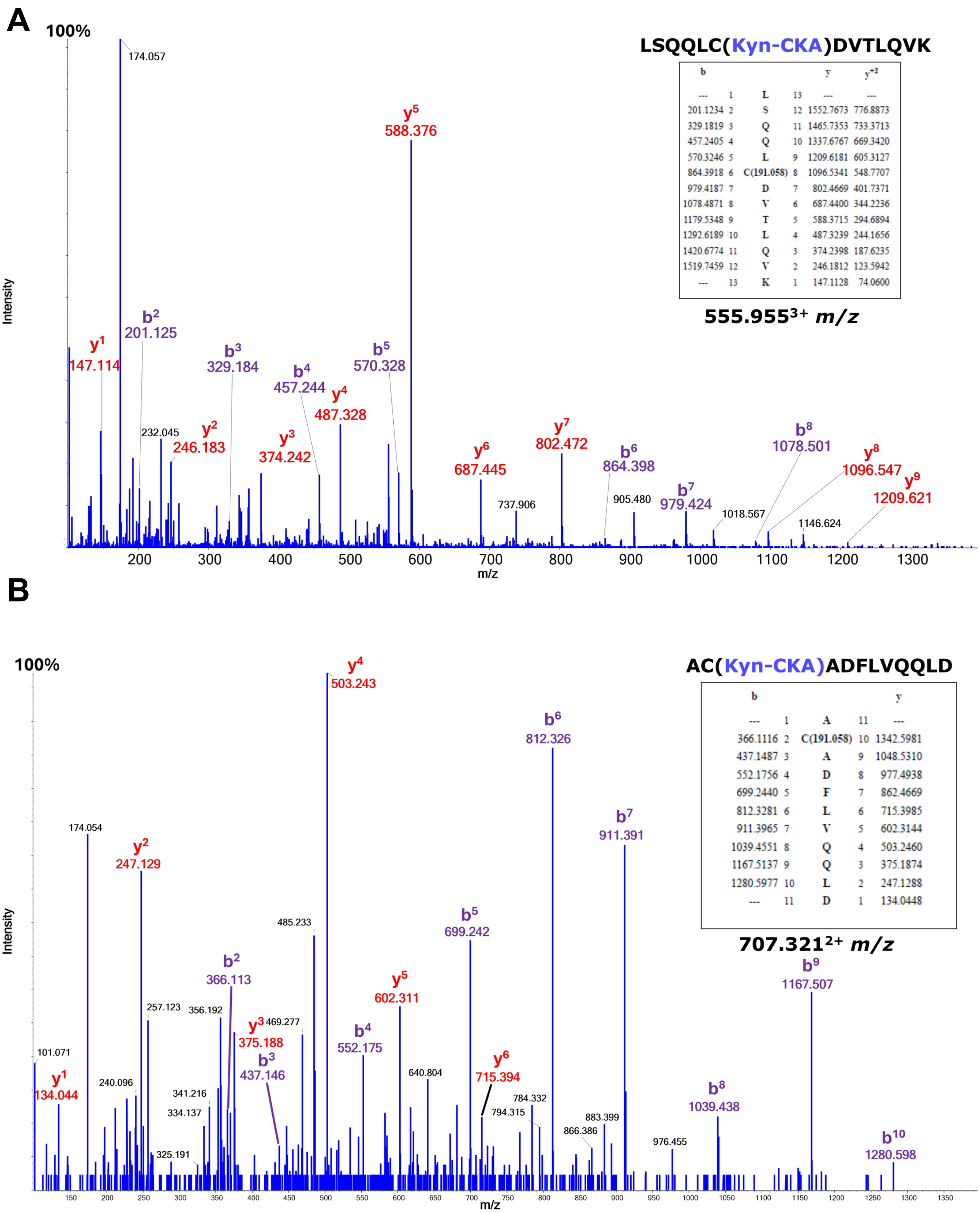

Supplementary Figure 1. LC-MS/MS characterization of the addition products of Kyn-CKA and Keap1 BTB-C77 and -C171. (A) Collision-induced dissociation (CID) spectrum of LSQQLC(Kyn-CKA)DVTLQVK demonstrating the modification of Keap1-BTB-C77 by Kyn-CKA characterized by the b6 and y8 ions in the series. **(B)** Collision-induced dissociation (CID) spectrum of AC(Kyn-CKA)ADFLVQQLD demonstrating the addition of Kyn-CKA to Keap1-BTB-C171 on the b2 ion. Kyn-CKA modification: m/z 191.058.
